# Metabolites extracted from browntop millet (Urochloa ramosa (L.) T.Q. Nguyen) mitigated the proliferation of breast- and colorectal carcinoma cells *in vitro* and retarded the EAC tumors *in vivo* by binding to oncogenic proteins

**DOI:** 10.3389/fphar.2026.1805287

**Published:** 2026-05-07

**Authors:** Mahadevaswamy G. Kuruburu, Venugopal R. Bovilla, Rimshia Naaz, Zonunsiami Leihang, Sahana Shivaramakrishna, P. Pramod Kumar, K. V. Harish Prashanth, Tousif Ahmed Hediyal, Dugganaboyana Guru Kumar, Saravana Babu Chidambaram, T Durai Ananda Kumar, Danielle Twilley, Namrita Lall, Preethi G. Anantharaju, SubbaRao V. Madhunapantula

**Affiliations:** 1 Center of Excellence in Molecular Biology and Regenerative Medicine (A DST-FIST Supported Center), Department of Biochemistry (A DST-FIST Supported Department), JSS Medical College, JSS Academy of Higher Education & Research (JSS AHER), Mysuru, Karnataka, India; 2 Department of Biochemistry, School of Life Science, JSS Academy of Higher Education & Research (JSS AHER), Mysuru, Karnataka, India; 3 Department of Biochemistry, CSIR-Central Food Technological Research Institute, Mysore, Karnataka, India; 4 Department of Pharmacology, JSS College of Pharmacy, JSS Academy of Higher Education & Research, Mysuru, Karnataka, India; 5 Centre for Experimental Pharmacology and Toxicology, JSS Academy of Higher Education & Research, Mysuru, Karnataka, India; 6 Department of Pharmaceutical Chemistry, JSS College of Pharmacy, Ooty, Tamil Nadu, India; 7 Department of Plant and Soil Sciences, Faculty of Natural and Agricultural sciences, University of Pretoria, Pretoria, South Africa; 8 School of Natural Resources, University of Missouri, Columbia, MO, United States; 9 Bio-Tech Research and Development Institute, The University of the West Indies, Mona, Jamaica; 10 Special Interest Group in Cancer Biology and Cancer Stem Cells (SIG-CBCSC), JSS Medical College, JSS Academy of Higher Education & Research (JSS AHER), Mysuru, Karnataka, India

**Keywords:** breast cancer, browntop millet, colon cancer, EAC, phenolics extract

## Abstract

Epidemiological studies have demonstrated an inverse correlation between millet-based diets and incidence of cancers of colon and rectum. These findings highlight the potential of millets as functional foods for effective management of cancers. But the experimental evidences supporting these observations remain limited. Among diverse millet varieties, Browntop millet (BTM) has recently been reported to possess potent anti-oxidant and DNA protection properties. Therefore, in the current study we have hypothesized that metabolite rich fractions of browntop millet, particularly the free metabolites extract (BTM-F), exert anticancer effects by targeting key oncogenic pathways that regulate cell proliferation, cell cycle progression, and apoptosis in breast and colorectal cancers. To test this hypothesis, the study aimed to (a) chemically characterize free and bound metabolites (BTM-F and BTM-B) using UPLC-QTOF-MS; (b) evaluate their anticancer efficacy *in vitro* and *in vivo* models; (c) delineate their effects on cell cycle regulation and apoptosis, including modulation of caspase-dependent pathways; and(d) investigate the molecular interactions of identified bioactive metabolite with selected oncogenic protein targets using *in silico* approaches. Results of the study showed that metabolites of browntop millet contained bioactive phenolics and flavonoids which possessed anticancer potential. Both BTM-F and BTM-B demonstrated significant antioxidant activity and reduced viability of breast- and colorectal cancer cells (IC_50_: 15.36 ± 0.86 to 67.44 ± 7.46 μg/mL). Mechanistically, BTM-F could induce G2/M cell cycle arrest and promoted apoptosis through increased caspase-3 expression and DNA fragmentation. *In silico* analyses revealed strong binding of key metabolites-Formononetin-7-O-glucuronide, Naringenin-7-O-β-D-glucuronide, and Apigenin-7-glucoside to COX-2 and mtALDH-2. *In vivo*, BTM-F but not BTM-B significantly suppressed EAC tumor growth. In summary, results of this study for the first time, demonstrated the anti-tumor properties of browntop millet free metabolites *in vitro* and *in vivo*. Future studies shall consider BTM-F for testing against patient-derived xenografts and develop functional food-based therapeutic formulation for the better treatment of cancers.

## 1 Introduction

Millets, also known as “Nutricereals”, have gained substantial recognition due to their high nutritional attributes and health benefits (Hassan et al., 2021). These gluten free grains are a rich source of proteins, vitamins, minerals and bioactive molecules including polyphenols, flavonoids, etc (Mazumder et al., 2024). The United Nations General Assembly considered year 2023 as the International Year of Millets (IYoM) with an aim of spreading the health-beneficial effects of millets and millet-derived products (Meena et al., 2021). Growing experimental evidences have shown that millets are a rich source of antioxidant molecules, which play a crucial role in protecting cells against oxidative stress and chronic and acute inflammatory reactions (Kuruburu et al., 2022a). The health beneficial effects of millets include (a) reduction in glycemic index, (b) boosting of host immune system, (c) augmentation of cellular anti-oxidant potential and (d) correcting the dysregulated gut microbiome (Arya and Bisht, 2022). Although studies have demonstrated the antioxidant and anti-proliferative properties of various millets, such as finger millet and foxtail millet, the potential health benefits of Browntop millet (BTM) remain largely unexplored.

**FIGURE 1:**
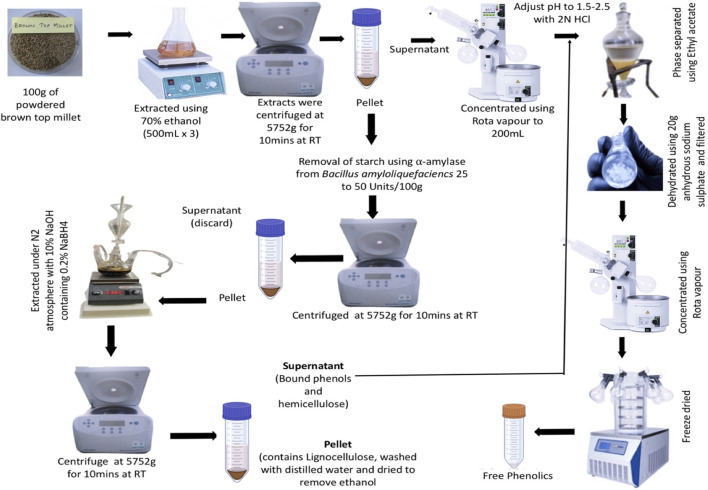
The pictorial flow-chart depicting the protocol implemented for extracting the free and bound secondary metabolites from browntop millet: Free phenolics were extracted using 70% ethanol at room temperature. The bound phenolics were isolated after digesting the starch followed by extraction with 10% sodium hydroxide. The extracts were concentrated using rotavapour and freeze-dried. The obtained extracts were dissolved with suitable solvents for further characterization and activity assays.

Browntop millet, scientifically known as *Urochloa ramosa* (L.) T.Q. Nguyen (Figure 1A) is a drought and heat-resilient crop originated in Southeast Asia. It is grown in Australia, China, Arabia, Western Asia, and Africa (Singh et al., 2022). Recent studies have reported variations in the physical attributes of BTM varieties that include differences in the moisture content, seed weight, seed volume, bulk density, and porosity (Nagaraju et al., 2020). Few other studies have revealed variations in the proximate composition of BTM varieties (Sravani et al., 2020), which consists of differences in the nutritional attributes (such as total protein, carbohydrate, fat and crude fiber) as well as the energy content of BTM (Sirisha et al., 2022). Studies have shown that various processing methods, genetic parameters, phenotypic attributes, geographical distribution and domestication traits are contributing to the differences observed in the proximate composition of BTM (Sravani et al., 2020) (Kingwell-Banham and Fuller, 2014).

Due to high fiber content BTM has been used in (a) reducing the complications of several metabolic disorders, (b) decreasing the constipation, (c) promoting effective detoxification, and (d) managing diabetes (Ashoka et al., 2020). In addition, the rich antioxidant content in BM assists in preventing gastric ulcers and colon cancers, while boosting the function of Central Nervous System (CNS), digestive-, skeletal- and immune systems (Singh et al., 2022). A recent study by Sunagar et al., 2023 evaluated the effect of different solvent polarities (acetone, ethanol, methanol and water) on the phenolic profiles and the role of extracted free phenols in regulating oxidative damage, DNA fragmentation, and inhibitory potential against α-amylase and α-glucosidase (Sunagar and Sreerama, 2023). Authors of this study have reported that phenolic compounds extracted using 80% acetone could protect DNA from oxidative damage and inhibited α-amylase and α-glucosidase activity more efficiently compared to other solvent extracts (Sunagar and Sreerama, 2023).

Despite its promising nutritional profile the specific effects of Browntop millet such as antioxidant and anticancer activities remain underexplored (Singh et al., 2022). Therefore, there is a need to conduct systematic research and establish pharmacological benefits using *in silico*, *in vitro* and *in vivo* models. With this background, the free- (BTM-F) and bound- (BTM-B) metabolite-rich extracts were prepared from whole BTM flour and characterized by UPLC-QTOF-MS. Formononetin-7-O-glucuronide, Naringenin-7-O-β-D-Glucuronide, and Apigenin-7-glucoside were among the various metabolites identified in BTM-F with known anti-cancer activity. *In silico* studies have shown that these metabolites strongly bind to cyclooxygenase-2 (COX-2) and mitochondrial aldehyde dehydrogenase-2 (mtALDH-2), which might be responsible for the anticancer activity exhibited by BTM-F. *In vitro* studies have shown that BTM-F is selective to cell lines representing carcinomas of breast (BT-474, MCF-7, MDA-MB-231 and MDA-MB-468), and carcinomas of colon and rectum (HCT-15, HCT-116 and HT-29), but not to normal skin keratinocyte cell line HaCaT. In mice, we have shown that oral administration of BTM-F retarded the development of Ehrlich Ascites Carcinomas (EAC). To the best of our knowledge, this is the first report comprehensively demonstrating the anticancer activity of phytochemical-rich extracts of Browntop millet.

## 2 Materials and methods

### 2.1 Materials

Breast cancer cell lines MCF7 (ER^+^, PR^+^ and HER2^2-^), MDA-MB-231 (ER^−^, PR^−^ and HER^2^) and MDA-MB-468 (ER^−^, PR^−^ and HER^2-^); Colorectal carcinoma cell lines HCT-15, HCT116 and HT-29 were procured from the National Center for Cell Science (NCCS) (Pune, Maharashtra, India). ER, PR and HER2 positive breast cancer cell line (BT-474) was provided by Dr. Annapoorni Rangarajan, Professor, Department of Molecular, Reproduction, Development and Genetics, Indian Institute of Science, Bengaluru, Karnataka, India. Human keratinocyte cell line (HaCaT) was provided by Dr. Gopinath, M.S. Principal Scientist, Department of Molecular Nutrition, CSIR-CFTRI, Mysore, Karnataka, India.

2,2-Diphenyl-1-picrylhydrazyl (DPPH), 2,3,5-Triphenyltetrazolium chloride (TPTZ) and Sulforhodamine B (SRB) were procured from Sigma Aldrich (St. Louis, MO, United States). Dulbecco’s modified Eagle’s medium (DMEM-high glucose 4.5 g/L), Foetal bovine serum (FBS), Glutamax, Trypsin, Dulbecco’s phosphate-buffered saline (DPBS) and Antimicrobial agents Penicillin and Streptomycin were procured from HiMedia Laboratories (Mumbai, Maharashtra, India). Cell culture plastic-ware, T25, T75, 15.0 mL and 50.0 mL conical tubes, disposable pipettes, micropipettes tips were procured from Techno Plastic Products (TPP) India, Pvt. Ltd, Bengaluru, Karnataka, India. All other reagents such as solvents, salts, acids and bases were of Analytical Reagent (AR) grade and procured from Sisco Research Laboratories (SRL), Pvt. Ltd., Mumbai, Maharashtra, India. Dimethyl sulfoxide (DMSO) and Caspase-3/7 Fluorescence assay kit were purchased from Cayman Chemicals (Ann Arbor, Michigan, United States). The BTM used in this study is authenticated and supplied by Krishi Vigyan Kendra (KVK), Mandya District, Karnataka, India. KVK is a part of University of Agricultural Sciences (UAS), Bangalore, Karnataka, India.

### 2.2 Methods

#### 2.2.1 Millet collection and processing

BTM seeds were washed with tap water to remove soil particles. The cleaned seeds were shade dried, powdered using a mixer-grinder (Preethi, Phillips Domestic Appliances India Ltd., Sholinganallur, Tamil Nadu, India) and the flour was stored at −80 °C for further use.

#### 2.2.2 Extraction of free and bound phenolic acids from browntop millet

Free and bound phenolic acids were extracted as described by Subba Rao and Muralikrishna (2002) with minor modifications. Free phenolic acids were extracted using 70% aqueous ethanol by continuous stirring at room temperature and the generated extract was concentrated using a rotary evaporator (Heidolph, Hei-VAP Core, Germany). The pH of the ethanolic extract was adjusted to ∼2.5 with 1N hydrochloric acid (HCl) and phase separated using ethyl acetate. The ethyl acetate phase was collected and dehydrated by the addition of anhydrous sodium sulphate (Na_2_SO_4_) (100 g/1000 mL of pooled ethyl acetate fraction). The dehydrated solution was filtered and concentrated using a rotary evaporator, followed by freeze-drying (Figure 1). The resulting extract was designated as BTM free metabolites extract (BM-F).

The residue, which remained after the extraction of free phenolics was used for the extraction of bound phenolic acids as detailed below. The pellet was de-starched using alpha-amylase (from *Bacillus amyloliquefaciencs* 25–50 Units/100 g dried residue) for 2–3 h at 80 °C. The de-starched material was subjected to the extraction of bound phenolics, which was carried out under continuous flow of nitrogen (N_2_) gas, with 10% sodium hydroxide (NaOH, 5 volumes) containing 0.2% sodium borohydride. After 2 h of extraction, the extract was centrifuged at 5752 Xg (Eppendorf 5430R Hamburg, Germany) and the supernatant containing bound phenolic acids, was collected. The residue, which predominantly contains lingo-cellulose, was washed thoroughly with distilled water to remove alkali, and air-dried. The supernatant, which contains hemi-celluloses as well as bound phenolic compounds was used to separate bound phenolic compounds. Procedurally, the pH of the supernatant was adjusted to ∼2 using concentrated HCl under cold conditions. Further processing was carried out as described in the extraction of free phenolic acids. The bound phenolic acid extract was designated as BTM bound phenolic extract (BTM-B) (Figure 1).

### 2.3 Determination of total phenolic content using the Folin-Ciocalteu reagent

The total phenolic content in the BTM-F and BTM-B was estimated using the Folin-Ciocalteu (FC) reagent as described by Sanchez Rangel et al., 2013. In brief, 70 µL of Gallic acid (concentration ranging from 2.5 μg/mL to 40 μg/mL), and suitably diluted BTM-F and BTM-B were aliquoted in to 96 well plate followed by the addition of 70 µL of FC reagent (diluted 1:1 with water) and 60 µL of 4.0% sodium carbonate. The reaction mixture was incubated for 30 min at room temperature. The absorbance was measured at 765 nm using a multimode plate reader (EnSpire2300, PerkinElmer Inc, MA, United States), and the concentration of phenolic content was calculated using the Gallic acid standard curve (Sánchez-Rangel et al., 2013).

### 2.4 Determination of total carbohydrate content by phenol sulfuric acid method

The total carbohydrate content of BTM-F and BTM-B was estimated as per the method described by Dubois et al., 1951. Briefly, 500 µL of glucose standards (concentration ranging from 10 μg/mL to 100 μg/mL) and diluted BTM-F and BTM-B were aliquoted into clean and dry glass test tubes and mixed with 500 µL of 5.0% phenol solution. Next, 2.0 mL of concentrated sulfuric acid was added and incubated at room temperature for 20 min. The absorbance was measured at 490 nm using a UV-Visible spectrophotometer (Bio Photometer 6131, Eppendorf, Hamburg, Germany). The concentration of total carbohydrate present in the samples was calculated using the glucose standard curve (Dubois et al., 1951).

### 2.5 Determination of reducing substances by dinitrosalicylic acid reagent

The content of reducing substances in BTM-F and BTM-B was determined by the dinitrosalicylic acid (DNS) method as described by Miller G.L. 1959. Briefly, 500 µL of glucose standards (concentrations ranging from 100.0 μg/mL to 800.0 μg/mL) and suitably diluted BTM-F and BTM-B were added to clean and dry glass test tubes and incubated with DNS reagent (prepared by mixing 75.0 mL of 6% sodium potassium tartrate with 30.0 mL of 5% DNS in 2M NaOH) for 10 min in a boiling water bath. Subsequently, 2 mL of distilled water was added to each tube, and the absorbance was measured at 540 nm using a UV-Visible spectrophotometer. The concentration of reducing substance in the samples was calculated using the glucose standard graph (Miller, 1959).

### 2.6 Determination of total protein content by bradford method

The total protein content in BTM-F and BTM-B was determined using the dye-binding method as described by Bradford et al., 1976. Briefly, 5.0 µL of Bovine Serum Albumin (BSA) standards (concentrations ranging from 50.0 μg/mL to 800.0 μg/mL) were incubated for 15 min with 250.0 µL Bradford reagent (prepared by dissolving 50.0 mg of Coomassie Brilliant Blue G-250 in 50.0 mL methanol and later mixed with 100.0 mL 85% phosphoric acid). The prepared reagent was diluted to 500.0 mL with water, mixed and filtered to remove precipitates. The filtered reagent was diluted to 1.0L by adding 350.0 mL of distilled water). The absorbance of the developed colour was measured at 595 nm using a UV-Visible spectrophotometer. The concentration of total protein (mg/gram extract) was calculated using the BSA standard curve (Bradford, 1976).

### 2.7 Determination of the antioxidant potential of BTM-F and BTM-B using ferric reducing antioxidant power (FRAP)

The FRAP assay was performed as reported earlier by Benzie IF and Strain JJ., 1999 (Benzie and Strain, 1999). Briefly, FRAP reagent was freshly prepared by combining 2.5 mL of 10 mM TPTZ (2,4,6-trypyridyls-triazine) (heated at 50 °C for 5 min), 2.5 mL of 20 mM ferric chloride, and 25 mL of 300 mM acetate buffer, pH 3.6. FRAP assay was carried out by mixing 10 µL of BTM-F and BTM-B (10–30 μg/mL total phenol content), or Gallic acid standard (in the range of 1–100 μg/mL) with 190 µL of FRAP reagent, followed by incubating the reaction mixture for 30 min at 37 °C. The absorbance of the developed blue colour was measured at 593 nm using a multimode plate reader (EnSpire2300, PerkinElmer, MA, United States). Subsequently, the absorbance values were converted to Gallic acid equivalent using the gallic acid standard curve.

### 2.8 Determination of DPPH radical scavenging activity

The radical scavenging potential of BTM-F and BTM-B was measured using the DPPH method according to Aksoy L et al., 2013 (Aksoy et al., 2013). Briefly, the reaction was performed by incubating 10 µL of BTM-F and BTM-B (10 μg/mL to 30 μg/mL total phenol content) with 140 µL of DPPH solution (6.2 mg in 100 mL of 100% ethanol) in the dark at room temperature for 30 min. Absorbance of the reaction mixture was measured at 536 nm using a multimode plate reader (EnSpire2300, PerkinElmer, Waltham, MA, United States). For construction of the standard curve, increasing concentrations of Gallic acid (5 μg/mL to 100 μg/mL) was used. The results were expressed as % free radical scavenging activity as shown below.

$$\text{Percentage free radical scavenging activity}=\left[A0-A1/A0\right]\times 100$$

A0 = Absorbance of DPPH reagent.

A1 = Absorbance of DPPH incubated with increasing concentrations of Gallic acid/Test sample.

### 2.9 Determination of nitric oxide (NO) radical scavenging capacity

The NO scavenging activity of BTM-F and BTM-B was determined using the method described by Mayur et al. (2010). Briefly 50 µL of serially diluted BTM-F and BTM-B (3 μg/mL to 50 μg/mL by total phenol content, in ethanol) was incubated with 50 µL of sodium nitroprusside (5 mM) for 90 min at room temperature and later mixed with 100 µL Griess reagent (N-(1-naphthyl) ethylenediamide dihydrochloride-0.1%, Sulfanilic acid-1% in 5% phosphoric acid). Gallic acid (125 μg/mL to 2000 μg/mL) was used as standard. The absorbance was measured at 546 nm using a BIO-TEK Power-Wave XS multi-well reader (Weltevreden Park, South Africa) using KC Junior Software. The fifty percent inhibitory concentration (IC50) was calculated using Graph Pad Prism 4 software.

### 2.10 Determination of the phytometabolites of BTM-F and BTM-B by UPLC-QTOF-MS

The phytochemical composition of BTM-F and BTM-B was analyzed using an Agilent 1290 Infinity LC system coupled with an Agilent 6500 Quadrupole Time-of-Flight (QTOF) mass spectrometer equipped with Agilent Jet Stream Thermal Gradient Technology. Stock solutions of BTM-F and BTM-B (1.0 mg/mL in methanol) were prepared and filtered through a 0.22 µm syringe filter prior to analysis. Chromatographic separation was performed on an Eclipse Plus C18 column (2.1 × 150 mm, 1.8 µm particle size) at a flow rate of 0.4 mL/min. The mobile phase consisted of 5 mM ammonium formate in water (A) and methanol (B), with the following gradient program: 0 min (2% B), 8 min (10% B), 12 min (20% B), 15 min (32% B), 20 min (40% B), 22 min (65% B), 26–28 min (95% B), 28.1 min (2% B), and 30 min (2% B). Mass spectrometric detection was carried out using electrospray ionization under optimized conditions. Data was acquired in full scan mode over a mass range of 100–1700 m/z with an acquisition rate of 4 spectra/sec. Metabolite identification was performed based on accurate mass measurements, MS/MS fragmentation patterns, and comparison with spectral databases and literature, following the criteria described by Kuruburu et al. (2022a).

Data Processing and Metabolite Identification:

Raw LC–MS data were processed using Agilent Mass Hunter Profinder software. Molecular features were extracted followed by binning and filtering of metabolite to remove noise and redundant signals. Data quality was ensured through QC-based assessment and normalization procedures. Subsequently, metabolites were grouped across samples based on retention time and mass alignment. Metabolite identification was carried out using accurate mass, isotopic distribution, and MS/MS fragmentation patterns in comparison with available databases and literature.

### 2.11 Determination of the anticancer activity of BTM-F and BTM-B

Anticancer activity of BTM-F and BTM-B was determined according to the method described by Madhunapantula et al. (2008). Briefly, 1 × 10^4^ cells per well (MDA-MB-231, MDA-MB-468, MCF7, BT-474, HCT-15, HCT 116, HT-29, and HaCaT) were seeded in a 96-well plate in 100 µL DMEM supplemented with 10% FBS, 2.5 mM Glutamax and 100U/mL of Pen-Strep, and allowed to grow for 36 h in an incubator (Forma 371 Steri Cycle CO_2_ Incubator, Thermo Fisher Scientific, MA, United States) set at 37 °C and 5% CO_2_. Exponentially growing cells were exposed to increasing concentration of BTM-F and BTM-BP (5 μg/mL to 80 μg/mL phenolic concentration) or the positive controls Diallyl disulphide (DADS, 1 mM) for 48 h. The number of viable cells was determined by SRB assay as described by Skehan et al., 1990 (Skehan et al., 1990). IC50 values were calculated using Graph Pad Prism 5.0 software.

### 2.12 Determination tumor growth inhibition of BTM-F by using Ehrlich Ascites Carcinoma (EAC) solid- and liquid tumor models

#### 2.12.1 EAC liquid tumor study

EAC liquid tumor study: Experiments in mice were performed after receiving the approval from the Committee for the Purpose of Control and Supervision of Experiments on Animals – (CPCSEA) approved Institutional Animal Ethics Committee (IAEC) of JSS Academy of Higher Education & Research (JSS AHER) (IAEC/008/2019). The efficacy of BTM-F to inhibit Ehrlich Ascites Carcinoma (EAC) cells proliferating in the ascites fluid of a mouse was assessed by following the protocol detailed by Bovilla et al. (2021). Female Swiss albino mice (5–7 weeks old) weighing approximately 25–30 g were divided into 5 groups. Group −1: BTM-F 200 mg/kg (n = 6); Group – 2: BTM-F 400 mg/kg (n = 6); Group – 3: Cisplatin (2.5 mg/kg) (n = 6); Group – 4: Tumor control (n = 8) and Group – 5: No tumor control group (n = 3).

**FIGURE 2:**
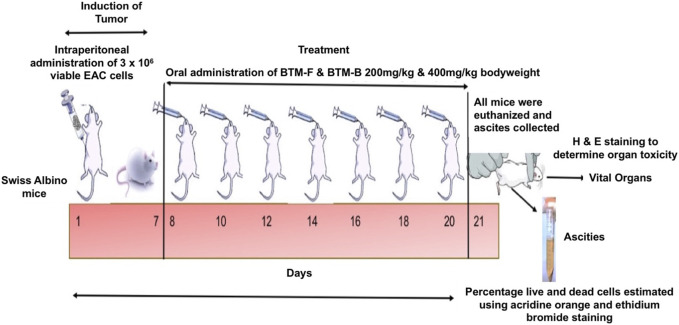
Experimental Design for Liquid Tumor Kinetics: Experimental mice were injected intraperitoneally with 3 × 10^6^ viable EAC cells as detailed in methodology section. BTM-F and BTM-B were administered orally at doses of 200 and 400 mg/kg body and cisplatin (2.5 mg/kg) was used as a positive control. Body weight, an indicator of tumor progression, was monitored every 2 days, and on day 21 mice were sacrificed for measurement of ascitic fluid volume and viable/dead EAC cell counts. Vital organs were subjected to H&E staining to assess tissue architecture and toxicity relative to control animals.

Procedurally, 3 × 10^6^ viable EAC cells (as determined by trypan blue exclusion method described by Strober (2001)) were injected into Intra peritoneal cavity of the experimental mice. The body weight of the mice was measured once every 2 days. BTM-F and BTM-B at a dose of 200 mg/kg and 400 mg/kg body weight were administered orally from 7^th^ day onwards till day 21 as detailed in Figure 2. These doses were selected based on a pilot acute toxicity study conducted as per Organization for Economic Cooperation and Development (OECD) guideline 425 (data not shown). Cisplatin (2.5 mg/kg) was used as positive control for tumor growth inhibition. The body weight, which is an indicator of tumor growth, was measured before injecting the drug at each administration time point. On the 21st day, all mice were sacrificed and the volume of collected ascitic fluid was measured. In addition, percentage live and dead EAC cells from each group was measured using the trypan blue exclusion method according to Strober et al., 2001. The vital organs (heart, kidney, liver and intestine) were collected and processed for Haematoxylin and Eosin (H&E) staining to evaluate the morphology and tissue architecture. Any visible variation in the vital organs tissue architecture or morphology compared to control mice was considered toxic.

#### 2.12.2 EAC Solid tumor study

**FIGURE 3:**
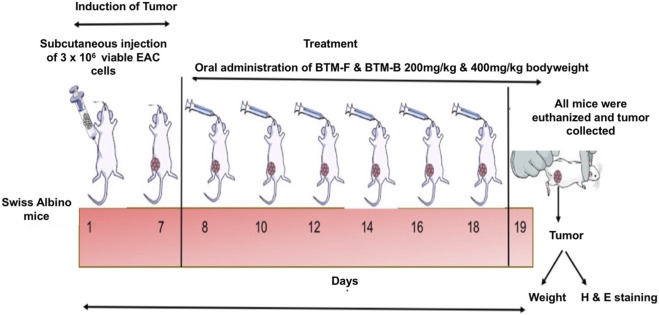
Experimental Design for Solid Tumor Kinetics: Experimentally 3 × 10^6^ viable EAC cells were injected subcutaneously into the thigh tissue of the mice. BTM-F and BTM-B were administered orally at doses of 200 and 400 mg/kg body and cisplatin (2.5 mg/kg) was used as a positive control. On day 19 mice were sacrificed and the tumors were collected and weighed. Vital organs were subjected to H&E staining to assess tissue architecture and toxicity relative to control animals.

The solid tumor study was carried out as described by Thirusangu et al. (2017). Briefly, female Swiss albino mice (5–7 weeks old) weighing approximately 26–30 g were divided into 5 groups viz., (1) BTM-F 200 mg/kg (n = 6), (2) BTM-F 400 mg/kg (n = 6) and (3) Cisplatin (2.5 mg/kg) (n = 6), (4) Tumor control (n = 8) and (5) No tumor control group (n = 9). Next, 3 × 10^6^ viable EAC cells were injected subcutaneously into the thigh tissue of experimental animals. The tumor volume was measured once every 2 days. Starting from the 8^th^ day, the mice were orally administered with BTM-F (200mgKg body weight and 400 mg/kg body weight) and the positive control Cisplatin (2.5 mg/kg body weight). BTM-B was not used in this study as it did not exhibit potent reduction in tumor growth when evaluated in the liquid tumor study. BTM-F and Cisplatin were administered every other day, and the treatment continued until the 18th day. The body weight was measured before injecting the drug at each administration. On the 19th day, all the mice from each group were humanely sacrificed, and the tumors were collected and weighed (Figure 3). The scarification of mice was performed as per the protocol described by CPCSEA. In brief, the mice were exposed to CO_2_ (CO_2_ asphyxiation) followed by applying the secondary method (i.e., cervical dislocation) of scarification to ensure the irreversible sacrification. The tumors from all groups were collected and processed for H&E staining to evaluate the morphology and tissue architecture.

### 2.13 Analysis of cell cycle by a 2-step 4′,6-diamidino-2-phenylindole (DAPI) staining procedure

Cell cycle analysis was conducted using the NucleoCounter® NC-3000 system as detailed by Beberok et al. (2018). Briefly, 1 × 10^6^ cells in 3.0 mL DMEM were seeded in 6-well plates and exposed to increasing concentrations of BTM-F and BTM-Bgg for 48 h. The treated cells were trypsinized, washed twice with DPBS by centrifugation at 500 X g for 10 min, and fixed by adding 4.5 mL of ice-cold 70% ethanol. The fixed cells were centrifuged at 500 X g for 5 min. The supernatant was carefully discarded and the collected cell pellet was washed with 5.0 mL PBS by centrifugation at 500 X g for 5 min. The cell pellet was re-suspended in 500 µL solution 3, which contains 1.0 μg/mL DAPI and 0.1% triton X-100 in PBS for 5 min at room temperature. An aliquot of 30 µL was loaded into the NC-Slide A2 and analyzed using Fixed Cell Cycle-DAPI assay program of NC 3000 (Beberok et al., 2018). The data was analyzed based on the number and intensity of DAPI stained cells. Histograms were captured and percentage cells in each cell cycle stage were determined. Cisplatin (50.0 µM) was used as a positive control.

### 2.14 Determination of cell death by staining with acridine orange (AO) and ethidium bromide (EtBr)

Cell death analysis was carried out by staining the cells with ethidium bromide (EtBr) and Acridine orange (AO) as detailed by Liu et al. (2015). Briefly, control and treated cells (0.5 × 10^6^) were trypsinized and mixed gently to obtain a single cell suspension. The trypsin was neutralized by the addition of DMEM supplemented with 10% FBS, and the cell suspension centrifuged at 1000 X g for 5 min. The obtained cell pellet was resuspended in 20 μL DPBS and incubated with 10.0 μL ethidium bromide (100.0 μg/mL in DPBS) and 10.0 μL acridine orange (100.0 μg/mL in DPBS) mixture for 10 min. The stained cells were imaged using an Olympus fluorescence microscope (BX53, Olympus, Tokyo, Japan) equipped with TRITC (red) and FITC (green) filters. The individual images from each filter were merged to obtain a combined image showing green (live), orange-red (apoptotic) and red (necrotic) cells (Chikkegowda et al., 2021).

### 2.15 Estimation of caspase-3/7 activity using a fluorophore-conjugated DEVD

#### 2.15.1 substrate

The caspase-3/CPP32 Fluorimetric assay kit (Cayman, cat#10009135) was used to measure the caspase-3/7 activity. Briefly, 0.5 × 10^6^ MDA-MB-231, BT-474, HCT-15 and HCT-116 cells were treated with BTM-F (5.0 μg/mL to 80 μg/mL) for 48 h and cell lysates from the control and treated cells were collected using 100 μL of lysis buffer (provided in the kit). The collected lysates were centrifuged and the clear supernatant used to determine the total protein content using BCA method (Yalamati et al., 2015). Caspase-3/7 activity was determined by incubating 50 μg of total protein in a final volume of 50 μL. Next, 50 µL of caspase-3/7 substrate (N-Ac-DEVD-N′-MC-R110) was added and the reaction mixture was incubated at 37 °C for 30 min. The fluorescence was measured at an excitation of 485 nm and an emission of 535 nm using a multimode plate reader (EnSpire2300, PerkinElmer Inc, MA, United States) (Chikkegowda et al., 2021).

### 2.16 Network pharmacology

#### 2.16.1 Software and hardware configuration

Web-based network pharmacology tools were accessed in Windows 11, Intel R Core (TM) i5-1100H@ 3.10 GHz, 2.61 GHz processor with 64-bit operating system for the identification of genes and protein-protein interaction analysis.

#### 2.16.2 Identification of molecular targets for the selected ligands

**FIGURE 4:**
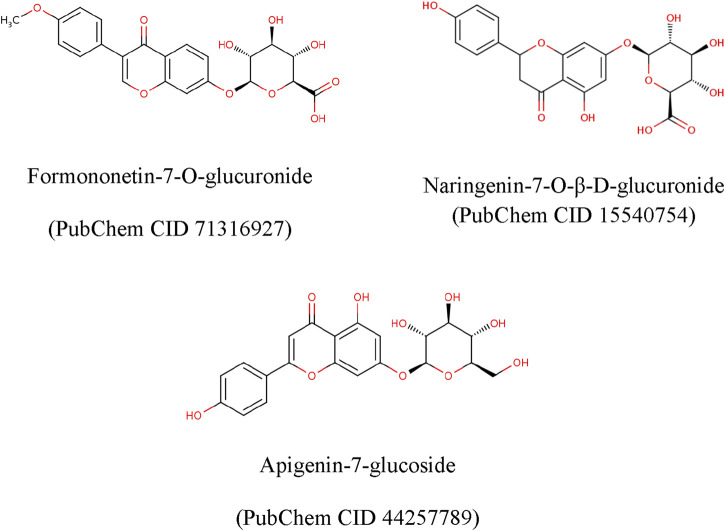
Structures of key metabolites identified in BTM-F that were subjected for molecular docking studies.

In order to identify the molecular targets for the selected ligands viz., formononetin-7-O- Glucuronide, Naringenin-7-O-β-D-Glucuronide, and Apigenin-7-Gucoside (Figure 4), a search was conducted by using “breast cancer” and “colorectal cancer” as keywords. The structural coordinates of ligands were retrieved from PubChem (https://pubchem.ncbi.nkm.nih.gov/). The targets and gene names for these ligands were retrieved from SwissTargetPrediction (http://www.swisstargetprediction.ch). The disease target genes of both cancers were collected from GeneCards (http://www.genecards.org) and DisGeNET (https://www.disgenet.org) using “Breast cancer” and “Colorectal cancer” as the search term.

#### 2.16.3 Visual analysis of targets and protein-protein interaction (PPI) network construction

Since, the protein-protein interaction analysis provides better understanding on the biochemical processes and their biological significances, the targets were imported into STRING database (https://string-db.org) and the intersection relationship was generated in accordance with “homosapiens”. The targets identified for the ligands were intersected with the cancer targets using Venn (https://bioinformatics.psb.ugent.be/webtools/Venn/). The generated network file (*.tsv) was imported from STRING database and analysed in Cytoscape v3.7.1. The confidence score >0.9 was considered in the target selection to minimize false positives. The ‘hub genes’, which play a key role in the PPI network were screened using the Cytohubba plugin (Szklarczyk et al., 2025).

### 2.17 Molecular docking

#### 2.17.1 Software and hardware configuration

The molecular docking simulations, binding energy calculations and ADMET prediction were performed in Schrodinger 2025–1 running on Dell precision 5820 workstation, Inter R Xeon (R) W-2255X20, 64 GB RAM, 3 TB HDD, Quadro RTX4000, Ubuntu 24.04LTS 64 bit, Windowing X11, Linux6.8.0–31 generic.

#### 2.17.2 Target preparation

X-ray crystallographic three-dimensional (3D) structures of target proteins cyclooxygenase-2 (COX-2, PDB ID: 3Q7D, 2.40 Å) and human mitochondrial aldehyde dehydrogenase (PDB ID: 2VLE, 2.40 Å) were downloaded from the Protein Data Bank (PDB, www.rcsb.org) (Berman et al., 2000). Target proteins were pre-processed and energy minimized with protein preparation wizard (OPLS4) of Schrödinger software 2025–1 (Trillmich et al., 1988). Protein structure steric conflicts were eliminated; gaps and splits were fixed utilizing prime software (Jacobson et al., 2004). The bond ordering was optimized, and hydrogen atoms were added. The water molecules away from 3 Å of catalytic pocket were deleted.

#### 2.17.3 Ligand preparation

The two-dimensional structures (*.sdf format) ligands, namely, formononetin-7-O-glucuronide (PubChem CID 71316927), naringenin-7-O-β-D-glucuronide (PubChem CID 15540754), and apigenin-7-glucoside (PubChem CID 44257789) were retrieved from PubChem database. Two-dimensional structure was used to convert into three-dimensional (3D) structure using OpenBabel software tool (O'Boyle et al., 2011). The energy of three-dimensional structures was minimized using the Steepest Descent (SD) algorithm in LigPrep module of Schrodinger. The structural neutralization (pH 7) and desalting of ligand was performed in Epik module based upon Hammett and Taft methodologies (Friesner et al., 2006).

#### 2.17.4 Docking simulations

The extra precision (XP Glide) docking algorithm was employed to predict the binding interactions of ligands and co-crystallized ligands against the target proteins. The XP Glide docking ranks ligands based on the binding ability to bind to the pocket residues The docking protocol was validated through redocking of co-crystallized ligand within the target active site (Eldridge et al., 1997). Euler angles, grid-based force field assessment, and Monte Carlo energy reduction were applied in the generation of ligand conformations (100 poses). Glide G-score, Glide H-bond, amino acid interactions and binding energy were considered to select the best pose of ligand within the binding pocket.

#### 2.17.5 Binding energy calculations

Molecular mechanics - Generalized Born Model and Solvent Accessibility (MM-GBSA) analysis for the ligand-receptor complexes were performed in Prime utilizing OPLS4 force field and rotamer algorithm (Lyne et al., 2006). The difference in free energy between the complex and the sum of the individual free energies of the ligand and target [ΔG_Bind_ = ΔG_Complex_ – (ΔG_Protein_–ΔG_Ligand_)] refers to binding free energy and describes the correlation between the experimental and predicted binding affinity.

### 2.18 Statistical analysis

Results of cytotoxicity assays were expressed as mean of three independent experiments, i.e., biological replicates, with at least 3 replicate measurements in each experiment, i.e., technical replicates, with ±standard error of mean (SEM). Graph Pad Prism version 5.0 (Graph Pad Software, CA, United States) was used for measuring the statistical significance between control untreated and experimental treated groups. The results (*In vitro*) were subjected to one-way analysis of variance (one-way ANOVA) to compare the differences between control and test groups and to determine the significance. Tukey’s *post hoc* test was applied. “*p*” value of <0.05*, <0.01** and 0.001*** was considered significant, highly significant and very highly significant, respectively. Similarly, differences in tumor volume at each time point were subjected to one-way ANOVA and significance calculated.

## 3 Results

### 3.1 Yield of free metabolites extract (BTM-F) is higher than bound metabolites extract (BTM-B)

Browntop millet free and bound metabolite extracts were prepared and the percentage yield was calculated (Supplementary Table ST1). Three independent extractions were conducted and the average percentage yield (grams per hundred gram of total flour) was found to be 0.496 ± 0.02 and 0.345 ± 0.04, respectively for BTM-F and BTM-B.

### 3.2 Composition analysis of BTM-F and BTM-B revealed the presence of phenolic acids, proteins, carbohydrates and DNS reacting substances with potent antioxidant activity

**FIGURE 5:**
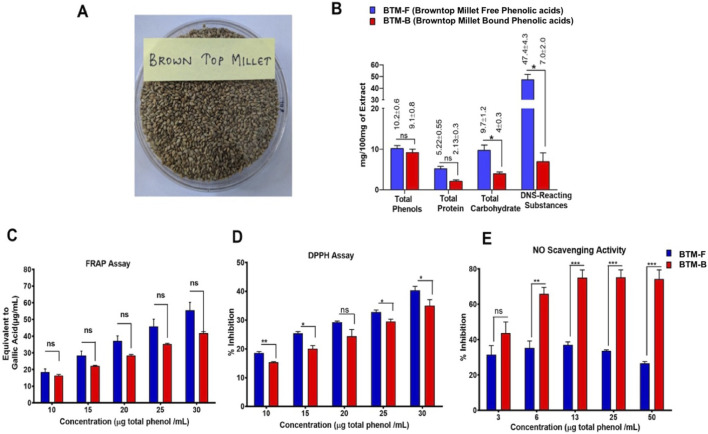
Compositional and antioxidant potential analysis of BTM-F and BTM-B: Browntop millet **(A)** BTM-F and BTM-B were found to contain total phenols, total proteins, carbohydrates and DNS reacting substances **(B)**. BTM-F and BTM-B showed dose-dependent ferric reducing antioxidant activity **(C)** and DPPH radical scavenging activity **(D)**. But no dose response was observed in NO radical scavenging activity in case of BTM-F; however, BTM-B exhibited a dose dependent NO radical scavenging till 13 µg total phenol/mL **(E)**.

The composition analysis of BTM-F and BTM-B was carried out by measuring the total carbohydrate, total protein, total phenol and DNS reacting substances. The phenolic content was 10.2 ± 0.6 mg/100 mg in case of BTM-F and 9.1 ± 0.8 mg/100 mg in case of BTM-B (Figure 5A). BTM-F was rich in DNS reacting substances (47.4% ± 4.3%), total protein (5.22% ± 0.55%) and total carbohydrates (9.7 %± 1.2%) compared to BTM-B, which had relatively low DNS reacting substances (7.0% ± 2.0%), total protein (2.13% ± 0.3%) and total carbohydrates (4.0% ± 0.3%) (Figure 5B; Supplementary Tables ST2A–S2D).

Since, prior studies have shown the ability of phenolic acid-containing extracts to exhibit antioxidant and radical scavenging activities, the antioxidant potential of BTM-F and BTM-B was evaluated by determining the ability to reduce Ferric ions (Fe^3+^) in to Ferrous ions (Fe^2+^) as well as their potential to scavenge the DPPH and NO free radicals. The BTM-F and BTM-B extracts showed dose-dependent antioxidant activity as depicted in Figures 5C–E. BTM-F exhibited higher ferric ion reducing activity and DPPH radical scavenging activity compared to BTM-B. But, BTM-B showed higher NO scavenging activity compared to BTM-F (Figures 5C,D; Supplementary Tables ST3A–S3D).

### 3.3 Characterization of BTM-F and BTM-B using UPLC-QTOF-MS showed the presence of hydroxy benzoic- and cinnamic acid derivatives

**FIGURE 6:**
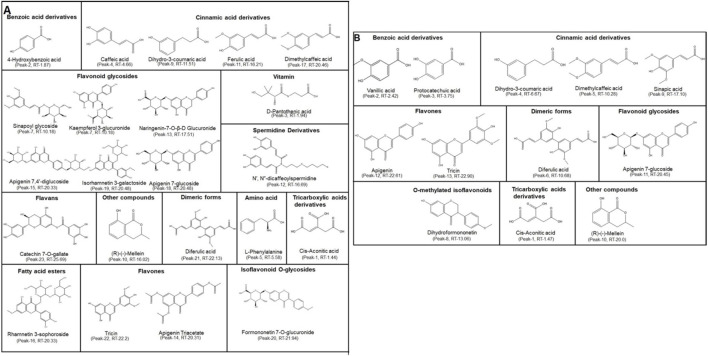
Phytochemical analysis of BTM-F and BTM-B using UPLC-QTOF-MS: In order to identify the phytochemicals present in the BTM-F and BTM-B, a UPLC-QTOF-MS analysis was performed, which showed the presence of phenolic compounds, flavonoids, flavones, tricarboxylic acid derivatives and amino acid derivatives along with several other metabolites **(A,B)**.

UPLC-QTOF-MS was used for the identification and relative quantification of secondary metabolites present in BTM-F and BTM-B (Kuruburu et al., 2022a). Analysis of the data showed the presence of 4-hydroxy benzoic acid, hydroxy cinnamic acid derivatives–caffeic acid, dihydro-3-coumaric acid, ferulic acid, dimethyl caffeic acid; flavonoid glycosides-sinapoyl glycoside, kaempferol 3-glucuronide, naringenin 7-0-ß-D glucuronide, apigenin 7,4′-di glucoside, isorhamentin 3-galactoside, apigenin 7-glucoside; flavones and flavans-tricin, apigenin triacetate, catechin 7-O-gallate; tricarboxylic acid derivative–cis-aconitic acid; diferulic acid; amino acid-phenylalanine and mellin (Figure 6A; Supplementary Figure SF1A; Supplementary Table ST4A) in the BTM-F extract.

The BTM-B extract was found to contain benzoic acid derivatives - vanillic acid and protocatechuic acid, cinnamic acid derivatives - dihydro-3-coumaric acid, sinapic acid and dimethyl caffeic acid; flavones–apigenin and tricin; diferulic acid; flavanoid glycosides - apigenin-7-glucoside; o-methylated isoflavonoid–dihydroformononetin; tricarboxylic acid derivative–cis-aconitic acid and mellin (Figure 6B; Supplementary Figure SF1B; Supplementary Table ST4B).

### 3.4 BMT-B reduced the viability of cancer cell lines

**FIGURE 7:**
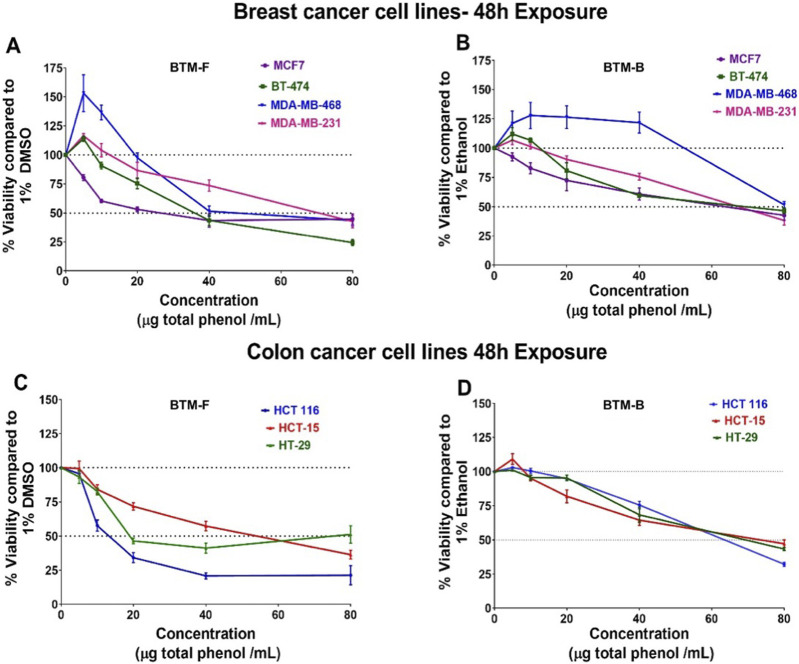
BTM-F and BMT-B reduced the viability of cancer cells: Dose-response curves of BTM-F and BTM-B against breast cancer cell line MDA-MB-231, MDA-MB-468, MCF7 and BT-474 **(A,B)**, colon cancer cell lines HCT 116, HCT-15 and HT-29 **(C,D)**.

Prior studies from our research group have reported the antiproliferative potential of hydroxy cinnamic and benzoic acid derivatives (Anantharaju et al., 2017). Based on these earlier studies, it was predicted that the phytochemical rich BM-FP and BM-BP could also exhibit anticancer activity. To test this, the effect of BTM-F and BTM-B on the viability of cell lines representing breast cancer and colon cancer was measured as detailed in methods section. Analyses of the data showed that both BTM-F and BTM-B decreased the viability of breast cancer cell lines (MDA-MB-231, MDA-MB-468, MCF7 and BT-474) (Figures 7A,B) and colon cancer cell lines (HCT 116, HCT-15 and HT-29) (Figures 7C,D) in a dose dependent manner. The selectivity index (SI) analysis of BTM-F and BTM-B showed variations in their selectivity as these extracts significantly reduced the viability of even the normal cell line HaCaT (Table 1). Further studies are warranted to reduce the toxicity against normal cells and improve the SI of these extracts.

**TABLE 1:** IC_50_ (µg/mL) value and selectivity index (SI) of BTM-F and BTM-B.

Cell line	IC50 values of BTM-F (µg total phenol/mL)	SI BTM-F vs. HaCaT	IC50 values of BTM-B (µg total phenol/mL)	SI BTM-B vs. HaCaT
MDA-MB-231	67.44 *± 7.46*	0.60	65.1 ± 5.87	0.99
MDA-MB-468	58.03 ± 6.45	0.70	76.65 ± 3.98	0.85
MCF7	30.1 ± 5.94	1.35	58.91 ± 10.73	1.10
BT-474	37.83 ± 2.44	1.07	63.16 ± 3.53	1.03
HCT 116	15.36 ± 0.86	2.6	60.67 ± 2.28	1.06
HCT-15	49.73 ± 5.03	0.81	68.2 ± 6.75	0.95
HT-29	45.11 ± 9.51	0.90	66.70 ± 3.12	0.97
HaCaT (normal keratinocyte cell line)	40.66 ± *3.11*	-	*64.84 ± 7.11*	-

### 3.5 BTM-F Retarded the Growth of EAC tumors in Mice

**FIGURE 8:**
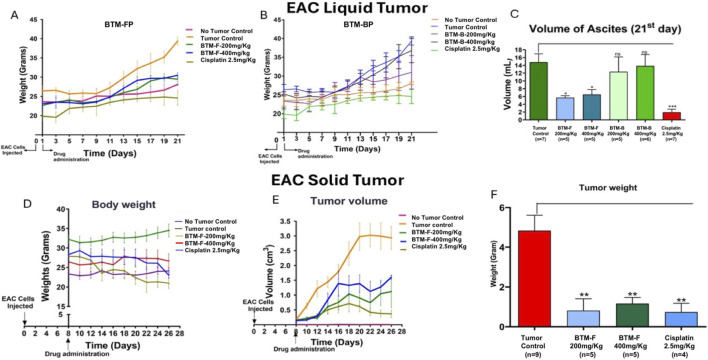
Oral BTM-F inhibited liquid and solid tumor growth in mice: Swiss albino mice, bearing liquid tumors were treated with BTM-F and BTM-B (200 mg/kg and 400 mg/kg) for a period of approximately 2 weeks. Cisplatin (2.5 mg/kg) was used as the positive control for tumor inhibition. Body weight was measured every second day **(A,B)**. Volume of ascites, collected on the 21st day, showed a significant decrease only in BTM-F treated mice but not in BTM-B treated mice **(C)**. Similarly, body weight and tumor volume were measured in every second day till the end of the study **(D,E)**. Approximately 80% decrease in the tumor weight was observed when mice bearing EAC solid tumors were treated with BTM-F **(F)**.

**FIGURE 9:**
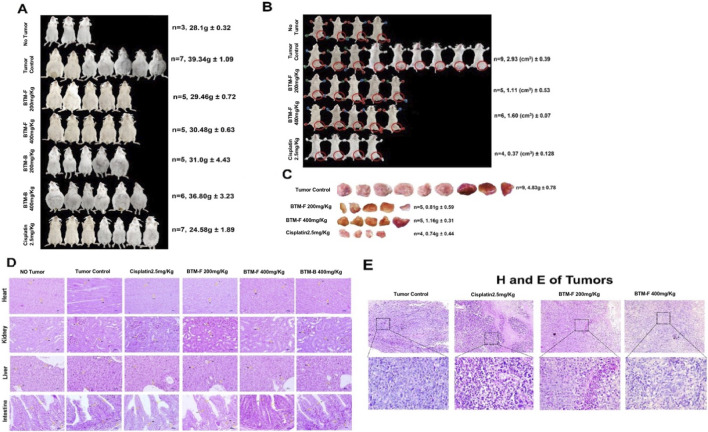
Analysis of vital organs of control and experimental mice, and the solid tumors: Photographs of the mice demonstrates the efficacy of BTM-F and the positive control cisplatin but not BTM-B. Note: A bulged abdominal region was observed in tumor control and mice treated with BTM-B, however no major changes (in abdominal region) in BTM-F treated mice were observed, which indicates the potential anti-tumor activity of this fraction **(A)**. Representative photographs of mice showed a significant reduction in tumor volume upon treatment with BTM-F. The volume of tumors (marked in circles, in mm^3^) with standard error, is shown in the photograph. Cisplatin was used as the positive control **(B)**, and reduction in the tumor weight was observed with the treatment of BTM-F **(C)**. To determine whether the administration of BTM-F or BTM-B had an impact on internal organs, heart, kidney, liver and intestine, these organs were fixed and sections analysed by staining with H and E. No significant changes in the tissue architecture and morphology were observed **(D,E)**.

Since the *in vitro* data showed anti-cancer activity of BTM-F, next we have assessed the impact of administering BTM-F in to mice bearing EAC tumors. The data showed a better cytotoxic potential against breast cancer cells *in vitro*. Next, the impact of these extracts on the growth of Ehrlic Ascites Carcinoma cells in the abdominal cavity of mice (liquid tumor model) was evaluated. Based on a pilot acute toxicity study (data not shown), doses of 200 mg/kg body weight and 400 mg/kg body weight were selected for the *in vivo* study. Oral administration of BTM-F, but not BTM-B, showed a significant decrease in the body weight of mice (an indicator of tumor growth) and ascites volume (another parameter indicating the tumor cells proliferation rates), compared to tumor control mice (Figures 8A–C). Cisplatin (2.5 mg/kg body weight), which was used as a positive control for tumor growth inhibition, reduced the ascites volume. Representative images of the mice showed a significant reduction in ascitic volume upon treatment with BTM-F (Figures 8D–F, Figure 9A). Under these experimental conditions, no changes were observed in the morphology and architecture of vital organs tissue sections (heart, kidney, liver and intestine) (Figures 9C,D).

Next, the potential of BTM-F for inhibiting the EAC solid tumors was evaluated. BTM-B was not used in this study as it showed minimal effect when tested in the liquid tumor model. Oral administration of BTM-F at 200 mg/kg and 400 mg/kg body weight showed a significant reduction in tumor growth compared to the control mice (Figure 9D). Growth reduction appeared from day 10 and continued till the end of the study (i.e., day 26; Figure 9E). A 4-fold decrease in the tumor weight was observed in the mice treated with 200 mg/kg BTM-F, which was almost equivalent to the positive control Cisplatin (2.5 mg/kg; Figure 9E). Representative images of the mice showed a significant reduction in tumor volume upon treatment with BTM-F (Figure 9B). The volume of tumors (marked in circles, in mm^3^) with standard error is shown in the photograph (Figure 9B).

### 3.6 Mechanistically, BTM-F and BTM-B extracts induced cell cycle arrest and apoptosis in breast- and colon cancer cell lines

**FIGURE 10:**
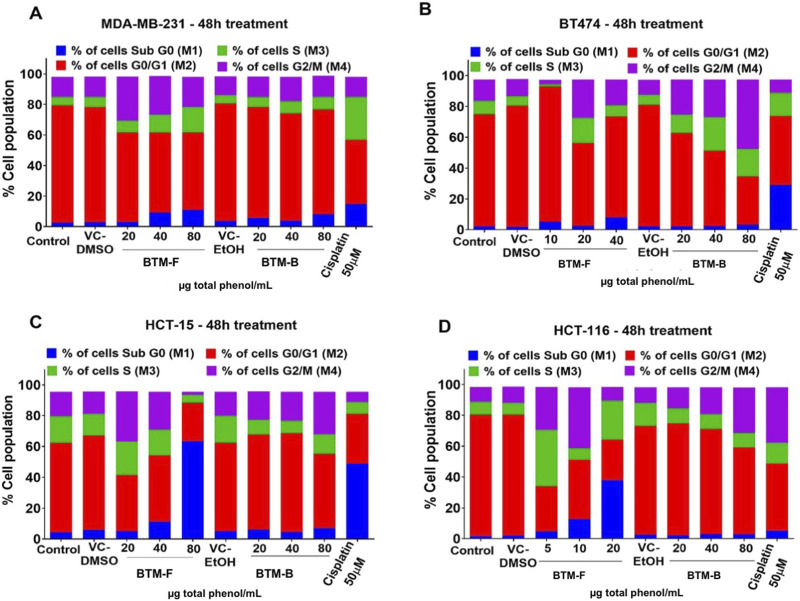
BTM-F and BTM-B modulated the cell cycle stages in breast and colon cancer cell lines: BTM-F and BTM-B differentially affected cell cycle stages in MDA-MB-231 **(A)**, BT-474 **(B)**, HCT-15 **(C)** and HCT-116 **(D)**. The extracts showed cell cycle arrest in G2/M phase and increased Sub G0/G1 phase.

Cell cycle arrest is one of the most common events triggered by majority of anticancer agents (Shapiro and Harper, 1999). Since BTM-FP and BTM-B could reduce the viability of breast and colon cancer cell lines, further analysis was carried out using MDA-MB-231 and BT-474 (breast cancer) and HCT 116 and HCT-15 (colon cancer) cell lines. Analysis of the data showed that BTM-F and BTM-B could induce G2/M cell cycle arrest in both breast and colon cancer cell lines (Figures 10A–D). Furthermore, a dose-dependent increase in sub G0/G1 population was observed in MDA-MB-231, HCT-15 and HCT 116 cells exposed to BTM-F (Figures 10A,B). The positive control Cisplatin arrested breast cancer cells in S-phase, however, a G2/M phase arrest was observed in case of colon cancer cell lines. An increase in the sub-G0 cell population was observed in all the cell lines (Figures 10C,D *)*.

**FIGURE 11:**
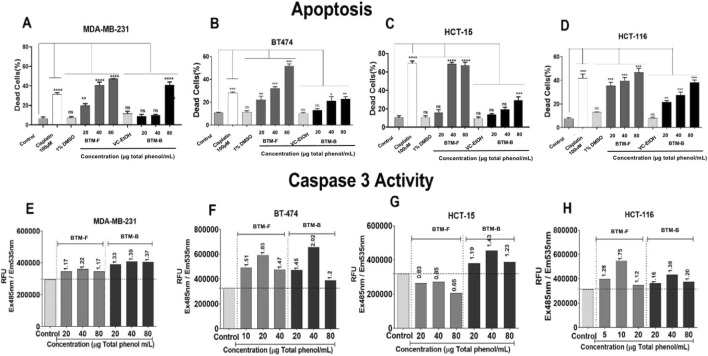
BTM-F and BTM-B induced apoptosis by Caspase 3/7 upregulation in breast- and colon rectal cancer cell lines: Breast cancer cell lines MDA-MB-231 and BT-474 and colon cancer cell lines HCT-15 and HCT 116 were exposed to increasing concentrations of BTM-F and BTM-B for 48 h and stained with ethidium bromide and acridine orange to observe apoptosis. A significant increase in the number of dead cells was observed upon treatment with BTM-F compared to BTM-B, specifically at 40 μg/mL in MDA-MB 231, BT-474 and HCT-15. HCT 116 showed an increase in apoptotic cells even at 20 μg/mL **(A–D)**. BTM-F effectively induced caspase-3 activity in BT-474 and HCT 116 cells but not in MDA-MB-231 and HCT-15 **(E–H)**.

Since elevated sub-G0/G1 phase cell population was observed in the MDA-MB-231, BT-474, HCT 116 and HCT-15 cells treated with BTM-F and BTM-B, we have presumed that these extracts might be inducing apoptosis through the induction caspases (Abou-Hashem et al., 2019). To confirm this, the control and treated cells were stained with acridine orange and ethidium bromide as explained in methods section (Figure 11). An increase in percentage of red cells, with increasing concentrations of the BTM-F and BTM-B was observed in all the four cell lines, indicating the induction of cell death, which was absent in the control cells (green cells). A 4-fold increase in the apoptotic cells was observed in MDA-MB-231 cells upon treatment with 40 μg/mL BTM-F, while BTM-B exhibited no significant increase in the dead cells at the concentrations tested (Figure 11A)*.* BT-474 cells were more susceptible to BTM-F as evident by a 5-fold increase in the dead cells compared to the control (Figure 11B). Although a dose dependent increase in dead cells was observed in BTM-B treated BT-474 cells it was not significantly higher when compared to the control cells even at highest concentration used (80 μg/mL). The positive control, Cisplatin (100 µM = 300 μg/mL) has also induced death in cancer cells tested (3-fold higher than control cells; Figures 11A,B). Among the colon cancer cell lines, HCT-15 was more susceptible than HCT 116 as evident by a 7-fold increase in the dead cell population at 40 μg/mL. A 4-fold increase in the dead cells was observed in HCT 116 cell line. BM-BP also exhibited dose dependent increase in dead cells in both HCT 116 and HCT-15 cells, but was not significantly higher when compared to BTM-F (Figures 11C,D).

To determine the mechanism by which BTM-F and BTM-B could induce cell death, we have measured the caspase-3 activity (an indicator of apoptotic cell death). Breast cancer cell lines MDA-MB-231 and BT-474, and colorectal cancer cell lines HCT 116 and HCT-15 were treated with BTM-F and BTM-B or the vehicle control for 48 h and the expression caspase-3 measured as mentioned in methods section. The data showed an elevated caspase-3 activity only in cell lines exposed to BTM-F but not in the vehicle control treated cells (Figures 11E–H). BTM-F could effectively induce caspase-3 activity in BT-474 and HCT 116 cell lines but did not induce caspase-3 activity to similar extent as observed in MDA-MB-231 and HCT-15 cells. BTM-B had minimal impact on caspase-3 activity in all the cell lines (Figures 11E–H).

### 3.7 Network pharmacology analysis identified key therapeutic targets modulated by the BTM-F constituents formononetin-7-O-glucuronide, naringenin-7-O-β-D-glucuronide and apigenin-7-glucoside

**FIGURE 12:**
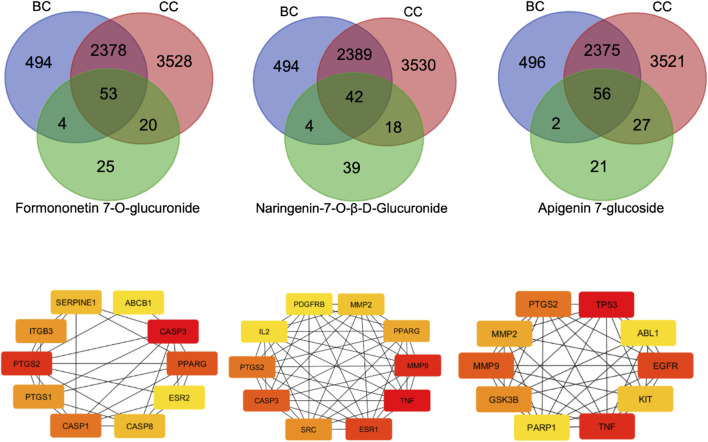
**(A)** Venn diagram and Cytoscape network constructions to identify genes intersection between ligands and cancer types. **(B)** Genes common to ligands and cancer types.

A total of 121 target genes were identified from SwissTargetPrediction database for the three ligands of BM-FP Formononetin-7-O-glucuronide, Naringenin-7-O-β-D-glucuronide and Apigenin-7-glucoside. Additionally, 2929 breast cancer genes and 5979 colorectal cancer genes were retrieved from GeneCards and DisGeNET with the species specification as “*Homo sapiens*” (Table 2). By merging the target genes of ligands, breast cancer and colorectal cancer, a Venn diagram was constructed (https://bioinformatics.psb.ugent.be/webtools/Venn/) (Figure 12A), which revealed 53, 42 and 56 common genes between the two cancers and three ligands respectively.

**TABLE 2:** Target genes identified for the ligands (PubChem CID 71316927, PubChem CID 15540754, PubChem CID 44257789).

Ligand	Gene codes
Formononetin 7-O-glucuronide	MMP2, XDH, CDK1, NQO2, GSTP1, PDGFRB, CA9, TYMS, HRAS, MME, MMP3, MMP8, MMP14, ABL1, CDK2, PDGFRA, HDAC1, GSTA1, MMP13, FOLR1, MAPK14, LDHA, CASP3, ACE, ITPR3, ALDH2, PIN1, DNMT3B, DHFR, TNF, ITGB1, KIT, PTGS2, ESR1, ENPP2, APEX1, MMP12, HMGCR, GLO1, GSTM1, SRC, MMP10, FOLH1, PPARG, ITGAV, DOT1L, IL2, CA2, ANPEP, ITGB3, PTGS,1 RPS6KA3, MMP9
Naringenin-7-O-β-D-Glucuronide	SHBG, CYP19A1, CASP1, TYMS, HRAS, PLA2G10, LGALS3, TYR, MMP13, RXRA, FOLR1, ABCB1, CASP7, POLB, CASP3, ABCG2, PTPN1, RNASEL, PRSS1, ITPR3, HSD17B1, CASP6, ALDH2, EDNRA, DHFR, PTGS2, SLC19A1, TERT, ABCC1, ESR2, CYP1B1, NRP1, PLA2G2A, CASP8, PPARG, ITGAV, EIF4A1, CA2, ANPEP, SERPINE1, ITGB3, PTGS1
Apigenin 7-glucoside	MMP2, ITGA5, XDH, CCNB1, MAOA, CDK1, CYP1A1, NQO2, KISS1R, CA9, GSK3B, EGFR, KCNA3, F10 ABL1, ODC1, PLG, CSNK2A1, PRKCD, ABCB1, PRKCE, ABCG2, CYP1A2, NQO1, SYK, CFTR, AR, HSD17B1, ALDH2, TNF, ITGB1, KIT, PTGS2, MMP12, GLO1, PRKCZ, TERT, TP53, PRKACA, ABCC1, CYP1B1, ALOX5, PLA2G2A, CDK6, ITGAV, ARG1, PFKFB3, PARP1, FLT3, IL2, CA2, SERPINE1, ITGB3 PRKCA RPS6KA3 MMP9

### 3.8 Protein-protein interaction network identified PTGS2 (cyclooxygenase-2, COX-2) and ALDH2 (mitochondrial aldehyde dehydrogenase 2) as the key targets of selected molecules

The protein–protein interaction network of ligand-caner was constructed in STRING. The Cytohubba plugin was used to identify the top ten genes in each network. A Venn diagram was drawn to find out the common genes associated with all the three ligands and cancers from the top 10 genes generated. The analysis indicated CA2 (carbonic anhydrase II), PTGS2 (cyclooxygenase-2, COX-2), ITGB3 (integrin beta-3/CD61), ALDH2 (mitochondrial aldehyde dehydrogenase 2), ITGAV (integrin alpha-V/CD51) as the common genes (Figure 12B; Table 3). In the narrow specification of targets, COX-2/PTGS2, and ALDH2 were identified as the targets involved in the biological activity of BM-FP. All the three ligands potentially act through these pathways to reduce the viability of breast cancer and colorectal cancer cells.

**TABLE 3:** Top five target genes and proteins identified for molecular docking simulations.

Gene symbol	Gene name	Protein name
CA2	Carbonic anhydrase 2	Carbonic anhydrase II
PTGS2	Prostaglandin-Endoperoxide Synthase 2	Cyclooxygenase-2 (COX-2)
ITGB3	Integrin Subunit beta 3	Integrin beta-3 (CD61)
ALDH2	Aldehyde dehydrogenase 2	Aldehyde dehydrogenase 2 (mitochondrial)
ITGAV	Integrin Subunit alpha V	Integrin alpha-V (CD51)

### 3.9 Molecular docking studies revealed the binding site and strength of selected molecules to identified targets

**FIGURE 13:**
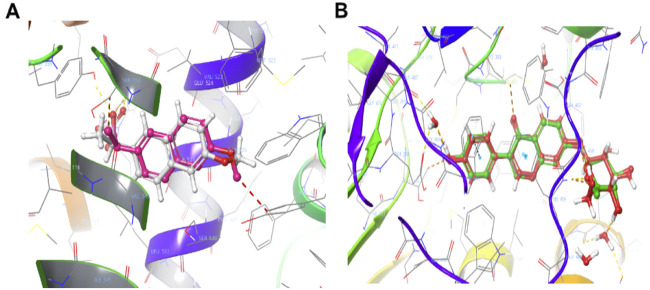
Superimposition of native cocrystal ligand and docked conformers. **(A)** 3Q7D (0.90 Å) **(B)** 2VLE (0.43 Å).

**FIGURE 14:**
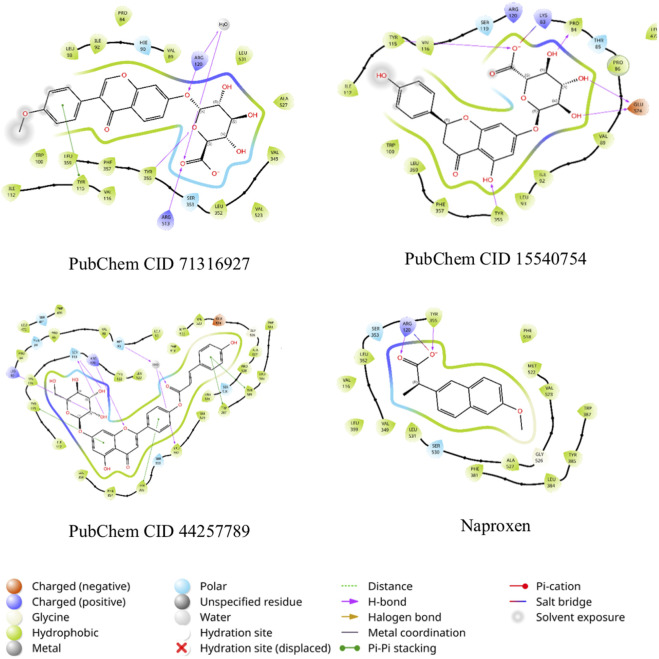
Two-dimensional (2D) interactions of PubChem CID 71316927, PubChem CID 15540754, PubChem CID 44257789 and Naproxen (co-crystal ligand) against 3Q7D.

The docking protocols were validated by superimposition of native and docked conformers against PDB ID: 3Q7D and 2VLE (Figure 13). The redocking of co-crystallized ligand within the target active site was used to validate the docking protocol, with the binding pose deviation <2 Å was considered as identical pose. The redocking of naproxen (co-crystal ligand) inside the binding pocket of 3Q7D produced a binding pose with an RMSD of 0.90 Å (limit <2 Å) (Figure 13). The carbonyl and the hydroxy moieties in the naproxen established an H-bond with the Arg120. The redocking of daidzin (co-crystal ligand) inside the binding pocket of 2VLE, 2.40 Å, produced a binding pose with RMSD of 0.43 Å (limit, <2 Å) (Figure 14). Val120 (-OH, 2.80 Å), Asp123 (-OH, 1.84 Å), Glu268 (-OH, 2.79 Å), Leu269 (-OH, 2.79 Å), Asp457 (-OH, 2.21 Å) are showing a hydrogen bond, and Phe459, Phe465 are showing π-π stacking interactions with the co-crystal ligand.

**FIGURE 15:**
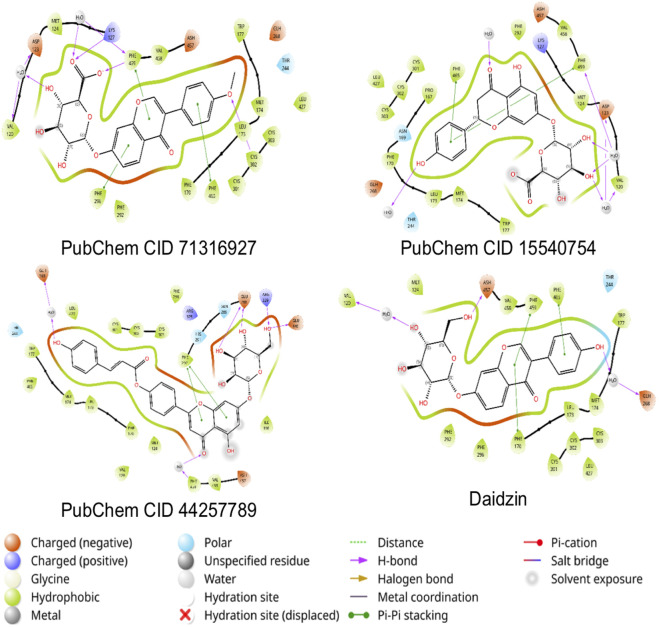
Two-dimensional (2D) interactions of PubChem CID 71316927, PubChem CID 15540754, PubChem CID 44257789 and daidzin (Co-crystal ligand) against 2VLE.

The ligands formononetin-7-O-glucuronide (PubChem CID 71316927), naringenin-7-O-β-D-glucuronide (PubChem CID 15540754), and apigenin-7-glucoside (PubChem CID 44257789) were docked against the 3Q7D.pdb and 2VLE.pdb grids generated around the cocrystal. The molecular docking simulation against 3Q7D produced glide score ranges from −6.05 to −10.91 kcal/mol. The compound 44257789 displayed higher XP-score (−10.91 kcal/mol) scores when compared to the cocrystal ligand (Table 4). Tyr355 π-π stacking and hydrophobic interactions are crucial in COX-2 inhibition, all the three ligands established the interaction (Figure 14) (Lowe et al., 2008). The molecular docking simulation results against 2VLE produced glide score ranges from −11.16 kcal/mol to −12.04 kcal/mol (Table 4). The compound 44257789 established interactions with Glu268, which is crucial for the 2VLE inhibition (Figure 15) (Duggan et al., 2011). Among the three metabolites, the compound 3 displayed higher XP- Score (−8.26 kcal/mol) and ΔG bind (−42.91 kcal/mol) scores compared with cocrystal ligand.

**TABLE 4:** Docking simulation and MMGBSA (ΔG bind) values for 3Q7D and 2VLE.

Comp. Code	XP and MM-GBSA computation (kcal/mol)			
	3Q7D		2VLE	
	XP- score	ΔG bind	XP- score	ΔG bind
Formononetin-7-O-glucuronide (PubChem CID 71316927)	−7.11	−1.54	−12.04	−84.85
Naringenin-7-O-β-D-glucuronide (PubChem CID 15540754)	−6.05	−23.67	−11.16	−68.59
Apigenin-7-glucoside (PubChem CID 44257789)	−10.91	−43.21	−11.93	−72.49
Co-crystal	−9.32	−11.62	−12.14	−89.44

### 3.10 Binding energy calculation

Prime Molecular Mechanics-Generalized Born Solvent Accessibility (MM-GBSA) analysis of post-docking minimized ligand-receptor complex illustrates the binding affinity through polar, hydrophobic and covalent bond interactions (Sastry et al., 2013). The binding free energies (ΔG bind) of the ligand poses are −1.54 and −43.21 kcal/mol, in 3Q7D. The post-docking minimized binding free energies (ΔG Bind) for 2VLE ranged from −68.59 to −84.85 kcal/mol, which are like the cocrystal values. The binding free energies (ΔG bind) of 44257789 in both the targets are comparable to that of cocrystal ligands (Table 4).

## 4 Discussion

Millets are often referred as “nutri-cereals”, and are known to exhibit antioxidant and anticancer properties (Zhang and Liu, 2015). Studies have also reported the presence of free sugars, amino acids, phenolic compounds, flavonoids and flavanones in the extracts of millets (Ratnavathi and Tonapi, 2022). Many preclinical and epidemiological studies have shown that diets rich in free sugars and total carbohydrates can promote cancer cells growth by altering the inflammatory and antioxidant pathways (Strober, 2001). But, recent studies have mentioned that the free sugars and total carbohydrates in the millet flours can help in reducing the tumor burden not only by enhancing gut health and metabolites that exhibit anticancer activity but also by sensitizing cancer cells to chemotherapeutic agents (Anand et al., 2008). In our study, we have observed that the BTM-F had more total carbohydrate, DNS reacting substances and protein compared to BTM-B. We have found that BTM-F is much potent in terms of reducing the viability of cancer cells, which could be in part due to the presence of excess total carbohydrates and sugars.

Numerous reports have also shown that the structure of plant secondary metabolites plays an important role in exhibiting antioxidant and anticancer activity (Singh et al., 2003). A review published by Anantharaju et al. (2016) summarised that phenolic compounds with increasing number of hydroxylic groups and with a double bond in the side chain exhibit better antioxidant and anticancer activity compared to the ones with–methoxy (OCH_3_) groups and or with no unsaturated side chain (Anantharaju et al., 2016). Previously, a study had isolated free- and bound phenolics from finger millet and correlated the antioxidant activity with the structure (Subba Rao and Muralikrishna, 2002). Several other studies from our research group have also shown the variations in antioxidant and anticancer activities of free and bound phenolic acids of finger- and foxtail millet and attributed the differences in activity to variations in their phenolic constituents. Authors of this research investigation have also mentioned the need for additional studies, as the extracts contain metabolites other than phenolic compounds and might be contributing to the anticancer and antioxidant properties (Kuruburu et al., 2022b). For example, a recent study has reported that peptides and derivatives of amino acids, which have been isolated from millets could exhibit antioxidant and anticancer properties (Shan et al., 2014). In correlation to these observations, the current study not only has shown the presence of several hydroxy benzoic- and cinnamic acid derivatives in the free and bound phenolic acids of browntop millet but also mentioned that the BM-FP had derivatives of amino acids and conjugates of phenolic compounds, which might be also contributing for the anticancer activity.

BTM-F was found to be rich in 4-hydroxy benzoic acid, caffeic acid and ferulic acid, while the BTM-B was rich in vanillic acid, protocatechuic acid and sinapic acid. In addition, both BTM-F and BTM-B were found to contain mellin and other secondary metabolites. Protocatechuic acid, caffeic acid and ferulic acid are known to exhibit potent antioxidant, anti-inflammatory and anticancer activities (Li and Yang, 2016). Similar to these observations many recent studies have also shown that millets rich in ferulic acid, caffeic acid, gallic acid and variety of flavonoids exhibit better anticancer potential against cancer cell lines (Duodu and Awika, 2019). For example, phytochemical extracts of Proso millet and Pearl millet that were rich in cinnamic acids have shown better anti-cancer activity against breast, colon and rectum and lung cancers (Zhang et al., 2014; Hithamani and Srinivasan, 2014). Similarly, a separate study has shown that fermented millet flours that were rich in cinnamic acids reported to kill the cancer cells more effectively than the unfermented millets (Dey et al., 2022). Furthermore, both BTM-F and BTM-B exhibited higher cytotoxicity against HCT 116 cells with an IC50 value of 15.36 µg total phenol/mL when compared to HCT-15 and HT-29 colon cancer cell lines. Among different breast cancer cell lines, MCF7 was found to be sensitive to BTM-F compared to MDA-MB-231, MDA-MB-468 and BT-474 cell lines. A study by Hajri et al., 2024 showed that the Pearl millet polyphenol-rich extract could inhibit triple negative, ER positive and HER2 positive cell lines *in vitro* (Hajri et al., 2024).

Even though Browntop millet extracts exhibited anti-cancer activity, they have also impacted the normal cell lines representing human keratinocytes suggesting that the selectivity of these extracts to cancer cells is minimal. Therefore, further studies to develop a formulation with enhanced selectivity are immediately required. One approach, which is widely used to reduce toxicity to normal cell while increasing the activity against tumor cells is to develop a nanoformulation (Lan et al., 2023). Supporting this strategy, a study has demonstrated that elagic acid loaded chitin nanoparticles could inhibit the viability of MCF-7 cells (Golmohammadi et al., 2023). Similarly, nanoencapsulated quercetin and curcumin exhibited better cytotoxicity against breast cancer cells compared to each of these drugs alone (Chen et al., 2020; Moon et al., 2021).

Millet and millet derived extracts have been shown to modulate the growth of tumors in animals (Zhang et al., 2020). Recently, a study has shown that the vanillin, isolated from Proso millet and Barnyard millet have induced cell cycle arrest and thereby reduced the tumor growth in mice (Ramadoss and Sivalingam, 2021). Similarly, another study showed that dietary supplementation of Foxtail millet extracts reduced colitis-associated colorectal carcinomas in animals (Shan et al., 2014). Numerous other studies have also documented the anticancer activity of millets and millet derived metabolites (Zhang et al., 2014; Singh et al., 2015). Mechanistically, millet extracts exert their effects by inducing cell cycle arrest, apoptosis and autophagic cell death (Xie et al., 2019). Similar to these observations, the results of the current study have demonstrated the anticancer activity of Browntop millet free phenolics in both liquid and solid tumor models. Since we have not observed any organ toxicity in mice, we recommend the usage of BTM-F for further development.

Deregulation of cell cycle stages and uncontrolled proliferation are commonly observed in cancer cells (Malumbres and Carnero, 2003). Recent studies have shown that phenolic compounds such as vanillic acid, gallic acid, protocatechuic acid, caffeic acid and genetisic acid and flavonoids such as apigenin and naringenin could inhibit colorectal cancer and breast cancer cell proliferation by inducing cell cycle arrest at G0-G1 phase (Abotaleb et al., 2020). A study conducted by Ujiki et al. (2006) showed that naturally occurring flavonoid Apigenin inhibited pancreatic cancer cells by inducing G2/M phase cell cycle arrest. Another study conducted by Xiong xiong Liu in 2023 showed that both apigenin and naringenin synergistically inhibited the proliferation of NSCLC by modulating cell cycle (Liu et al., 2023). Similarly, Kaempferol, which is another flavonoid, arrested breast cancer cell lines in the G2/M phase (Zhu and Xue, 2019), which corresponds to the current study wherein BM-FP and BM-BP rich in these bioactive molecules also arrested breast cancer cells in the G2/M phase and induced apoptosis as evident by an increase in the sub G0 population.

Caspases are important mediators of apoptosis (Thornberry, 1998). Among several caspases identified in the apoptotic signaling, caspase 3 appears to be activated in both intrinsic and extrinsic pathways, thus making it an important marker of apoptosis (Porter and Janicke, 1999). Phenolic compounds and flavonoid rich extracts induce apoptosis by regulating the caspase activity (Choi and Kim, 2009). A recent study reported by Wani et al. (2023) showed that nanoparticle-based phytochemicals induce the expression of caspase 3 and thereby modulate cancer cell proliferation. In line with these results, phytochemical rich BTM-F and BTM-B also induced the expression of caspase 3 in BT-474 and MDA-MB-231 cell lines.

Network pharmacology is one of the emerging areas of research, which assists not only in identifying key therapeutic targets but also helps in studying the interaction of pharmacological agents with the selected drug targets (Noor et al., 2022). In this study, we have identified COX-2 and ALDH2 as the targets that are commonly dysregulated in breast and colorectal cancers, and shown that the binding of key pharmacological agents of BTM-F viz., formononetin-7-O-glucuronide, naringenin-7-O-β-D-glucuronide, and apigenin-7-glucoside to these targets. Although further experimental validation is essential, we predict that these molecules effectively bind to the COX-2 and ALDH2 thereby retard the proliferation of tumor cells *in vitro* and *in vivo*. Formononetin is a potent anti-breast cancer agent. As a monotherapeutic or in combination with Metformin, Formononetin has been shown to inhibit the breast cancer cells (Xin et al., 2019). However, it is unknown whether Formononetin could inhibit oncogenic COX-2. Although a study has shown that Formononetin can activate ALDH2, it is unknown how the activation of ALDH2 reduces tumor cells viability.

Apigenin, which is another key molecule identified in our study, has been shown by many investigators that it binds to COX-2 and ALDH-2 thereby inhibit their activity (Kiraly et al., 2016). Although COX-2 is a proven therapeutic target in breast cancers, the involvement of ALDH-2 in breast and CRC require many in depth studies. Naringenin, another bioactive molecule identified through network pharmacology analysis, has been reported to exert significant growth-inhibitory effects against breast and colorectal cancer cells. Consistent with the *in silico* findings, previous studies have demonstrated that naringenin inhibits cyclooxygenase-2 (COX-2), thereby attenuating inflammation-driven tumor progression (Stabrauskiene et al., 2022). In addition, accumulating evidence indicates that its antiproliferative activity is largely mediated through modulation of the PI3K/AKT/mTOR signaling pathway, a central regulator of cancer cell survival, proliferation, and metabolism (Cheng et al., 2020).

## 5 Conclusion

This study provides the first evidence of the anticancer potential of browntop millet free metabolites extract (BTM-F) against breast- and colorectal cancers *in vitro* and *in vivo.* Phytometabolites analysis revealed the presence of benzoic- and cinnamic acid derivatives and flavonoids which are known to possess several pharmacological activities. Mechanistically, BTM-F induced G2/M cell cycle arrest and apoptosis via caspase-3 activation. *In vivo*, BTM-F effectively suppressed both liquid and solid EAC tumor growth up to 80%. Network pharmacology further indicated the role of flavonoid glucuronides-formononetin-7-O-glucuronide, naringenin-7-O-β-D-glucuronide, and apigenin-7-glucoside in modulating key oncogenic pathways. Notably, BTM-F exhibited superior efficacy compared to BTM-B both *in vitro* and *in vivo* models. These findings highlight BTM-F as a promising candidate for further development as a functional anticancer agent.

### 5.1 Limitation and future direction

The current study is limited to initial mechanistic insights focusing only on cell cycle arrest and caspase-3–mediated apoptosis, without detailed pathway analysis or broader apoptosis markers such as PARP or BCL2/BAX. Additionally, molecular docking predictions are not experimentally validated. Therefore, future studies shall focus on comprehensive pathway elucidation, expanded apoptosis profiling using proteome-level approaches, target validation, and testing in advanced tumor models to strengthen translational potential.

## Data Availability

The original contributions presented in the study are included in the article/Supplementary Material, further inquiries can be directed to the corresponding authors.
