# Supplementary material for: Metabolites extracted from browntop millet (Urochloa ramosa (L.) T.Q. Nguyen) mitigated the proliferation of breast- and colorectal carcinoma cells in vitro and retarded the EAC tumors in vivo by binding to oncogenic proteins
Source: Front Pharmacol. 2026 May 7;17:1805287. doi: 10.3389/fphar.2026.1805287 (PMC13190552; doi:10.3389/fphar.2026.1805287)
Supplement: Supplementary file 1 [file Supplementaryfile1.docx]

**SUPPLEMENTAL TABLES**

**Supplemental Table 1: Proximate composition analysis of three independent batches of BTM-F and BTM-B**

***ST-1A. Total Carbohydrate (mg/100mg extract; Glucose equivalent)***

|  | Batch-1 | Batch-2 | Batch-3 | Mean ± SE |
| --- | --- | --- | --- | --- |
| **BTM-F** | 7.53 | 11.8 | 10.04 | 9.79±1.24 |
| **BTM-B** | 3.3 | 4.37 | 4.33 | 4±0.35 |

***ST-1B. Total Phenol Content (mg/100mg extract; Gallic acid equivalent)***

|  | Batch-1 | Batch-2 | Batch-3 | Mean ± SE |
| --- | --- | --- | --- | --- |
| **BTM-F** | 8.861 | 10.74 | 11.051 | 10.22±0.68 |
| **BTM-B** | 8.031 | 8.71 | 10.83 | 9.19±0.84 |

***ST-1C. Total Protein (mg/100mg extract; BSA equivalent)***

|  | Batch-1 | Batch-2 | Batch-3 | Mean ± SE |
| --- | --- | --- | --- | --- |
| **BTM-F** | 4.61 | 6.33 | 4.73 | 5.22±0.55 |
| **BTM-B** | 2.73 | 1.71 | 1.95 | 2.13±0.3 |

***ST-1D. DNS Reacting Substances (mg/100mg extract; Glucose equivalent)***

|  | Batch-1 | Batch-2 | Batch-3 | Mean ± SE |
| --- | --- | --- | --- | --- |
| **BTM-F** | 40.12 | 46.94 | 55.24 | 47.43±4.37 |
| **BTM-B** | 11.16 | 4.87 | 5.01 | 7.01±2.07 |

**Supplemental Table 2. Antioxidant activity analysis of three independent preparations of BTM-F and BTM-B**

***ST-2A. FRAP of* BTM-F *(in µM Ferrous Sulfate)***

| **BTM-F** (µg Total Phenol / mL) | Batch-1 | Batch-2 | Batch-3 | Mean ± SE |
| --- | --- | --- | --- | --- |
| 10 | 386.26 | 293.70 | 429.87 | 369.94±4.19 |
| 15 | 581.64 | 481.79 | 676.80 | 580.08±5.37 |
| 20 | 778.82 | 647.68 | 868.74 | 765.08±6.26 |
| 25 | 941.35 | 790.87 | 1114.18 | 948.80±9.52 |
| 30 | 1101.64 | 1007.63 | 1349.10 | 1152.79±11.96 |

***ST-2B. FRAP of* BTM-B*(in µM Ferrous Sulfate)***

| **BTM-B**(µg Total Phenol / mL) | Batch-1 | Batch-2 | Batch-3 | Mean ± SE |
| --- | --- | --- | --- | --- |
| 10 | 237.00 | 321.13 | 316.29 | 291.47±7.30 |
| 15 | 350.13 | 428.63 | 473.43 | 417.39±6.08 |
| 20 | 441.38 | 560.50 | 606.29 | 536.05±9.21 |
| 25 | 576.38 | 696.75 | 763.43 | 678.85±4.80 |
| 30 | 607.63 | 834.25 | 889.86 | 777.24±8.42 |

***ST-2C. DPPH analysis of* BTM-F *(Vitamin-C equivalent)***

| **BTM-F** (µg Total Phenol/mL) | Batch-1 | Batch-2 | Batch-3 | Mean ± SE |
| --- | --- | --- | --- | --- |
| 10 | 17.77 | 18.92 | 19.24 | 18.64±0.45 |
| 15 | 25.41 | 24.35 | 26.43 | 25.39±0.60 |
| 20 | 29.56 | 29.75 | 28.34 | 29.22±0.44 |
| 25 | 32.41 | 31.65 | 34.16 | 32.74±0.74 |
| 30 | 37.58 | 42.00 | 41.39 | 40.32±1.38 |

***ST-2D. DPPH analysis of* BTM-B*(Vitamin-C equivalent)***

| **BTM-B**(µg Total Phenol/mL) | Batch-1 | Batch-2 | Batch-3 | Mean ± SE |
| --- | --- | --- | --- | --- |
| 10 | 14.88 | 15.29 | 15.83 | 15.33±0.28 |
| 15 | 17.92 | 20.97 | 21.30 | 20.06±1.08 |
| 20 | 19.65 | 27.18 | 26.25 | 24.36±2.37 |
| 25 | 28.18 | 29.62 | 30.85 | 29.55±0.77 |
| 30 | 30.92 | 37.58 | 36.50 | 35.00±2.06 |

**Supplemental Table 3. Cytotoxic potential of three batches of BM-FP and BM-BP**

***ST-3A. Effect of* BTM-F *against HCT-116 cells viability***

|  | Batch-1 | Batch-2 | Batch-3 | Mean ± SE |
| --- | --- | --- | --- | --- |
| Vehicle DMSO (1%) | 94.74 | 91.65 | 92.41 | 92.93±0.93 |
| DADS (1.0mM) | 57.02 | 67.83 | 55.84 | 60.23±3.82 |
| BM-FP (µg Total Phenol / mL) |  |  |  |  |
| 5 | 98.77 | 92.24 | 96.10 | 95.71±1.90 |
| 10 | 63.97 | 58.83 | 50.26 | 57.69±4.00 |
| 20 | 29.14 | 32.28 | 41.38 | 34.27±3.68 |
| 40 | 19.65 | 17.80 | 25.23 | 20.90±2.24 |
| 80 | 7.75 | 25.50 | 30.82 | 21.36±6.98 |

***ST-3B. Effect of* BTM-B *against HCT-116 cell line***

|  | Batch-1 | Batch-2 | Batch-3 | Mean ± SE |
| --- | --- | --- | --- | --- |
| Vehicle Ethanol (1%) | 94.39 | 98.65 | 96.65 | 96.56±1.23 |
| DADS (1.0mM) | 57.02 | 67.83 | 55.84 | 60.23±3.82 |
| BM-BP (µg Total Phenol / mL) |  |  |  |  |
| 5 | 104.59 | 101.79 | 102.27 | 102.89±0.87 |
| 10 | 98.78 | 98.53 | 104.07 | 100.46±1.81 |
| 20 | 93.76 | 93.66 | 96.68 | 94.70±0.99 |
| 40 | 74.74 | 71.14 | 80.61 | 75.50±2.76 |
| 80 | 31.28 | 30.20 | 35.23 | 32.24±1.53 |

***ST-3C. Effect of BTM-F* *against HCT-15 cells viability***

|  | Batch-1 | Batch-2 | Batch-3 | Mean ± SE |
| --- | --- | --- | --- | --- |
| Vehicle DMSO (1%) | 83.62 | 86.11 | 89.57 | 86.43±1.73 |
| DADS (1.0mM) | 41.25 | 50.15 | 47.52 | 46.31±2.64 |
| BM-FP (µg Total Phenol / mL) |  |  |  |  |
| 5 | 99.60 | 89.87 | 108.63 | 99.37±5.42 |
| 10 | 89.25 | 77.70 | 85.72 | 84.22±3.42 |
| 20 | 74.55 | 66.47 | 74.39 | 71.80±2.67 |
| 40 | 54.92 | 53.00 | 64.19 | 57.37±3.46 |
| 80 | 30.77 | 37.04 | 41.68 | 36.50±3.16 |

***ST-3D. Effect of BTM-B* *against HCT-15 cells viability***

|  | Batch-1 | Batch-2 | Batch-3 | Mean ± SE |
| --- | --- | --- | --- | --- |
| Vehicle Ethanol (1%) | 96.27 | 90.64 | 91.45 | 92.79±1.76 |
| DADS (1mM) | 41.25 | 50.15 | 47.52 | 46.31±2.64 |
| BM-BP (µg Total Phenol / mL) |  |  |  |  |
| 5 | 101.84 | 110.02 | 115.34 | 109.07±3.93 |
| 10 | 92.50 | 98.94 | 93.75 | 95.06±1.97 |
| 20 | 82.38 | 73.60 | 89.72 | 81.90±4.67 |
| 40 | 59.77 | 61.66 | 71.94 | 64.46±3.79 |
| 80 | 46.62 | 42.60 | 52.47 | 47.23±2.87 |

|  |
| --- |

***ST-3E. Effect of BTM-F* *against HT29 cells viability***

|  | Batch-1 | Batch-2 | Batch-3 | Mean ± SE |
| --- | --- | --- | --- | --- |
| Vehicle DMSO (1%) | 86.95 | 90.67 | 92.44 | 90.02±1.62 |
| DADS (1.0mM) | 52.99 | 59.40 | 49.35 | 53.91±2.94 |
| BM-FP (µg Total Phenol / mL) |  |  |  |  |
| 5 | 102.09 | 86.17 | 91.39 | 93.22±4.69 |
| 10 | 87.58 | 79.74 | 80.45 | 82.59±2.51 |
| 20 | 44.45 | 44.42 | 50.57 | 46.48±2.05 |
| 40 | 38.26 | 36.96 | 48.54 | 41.26±3.67 |
| 80 | 38.57 | 56.65 | 58.55 | 51.25±6.38 |

***ST-3F. Effect of BTM-B* *against HT29 cells viability***

|  | Batch-1 | Batch-2 | Batch-3 | Mean ± SE |
| --- | --- | --- | --- | --- |
| Vehicle Ethanol (1%) | 96.24 | 98.88 | 96.75 | 97.29±0.81 |
| DADS (1mM) | 52.99 | 59.40 | 49.35 | 53.91±2.94 |
| BM-BP (µg Total Phenol / mL) |  |  |  |  |
| 5 | 100.47 | 99.60 | 103.28 | 101.12±1.11 |
| 10 | 98.39 | 95.90 | 92.46 | 95.58±1.72 |
| 20 | 95.46 | 91.83 | 98.62 | 95.30±1.96 |
| 40 | 58.11 | 77.39 | 69.48 | 68.33±5.60 |
| 80 | 42.57 | 41.81 | 45.76 | 43.38±1.21 |

***ST-3G. Effect of BTM-F* *against MDA-MB-468 cells viability***

|  | Batch-1 | Batch-2 | Batch-3 | Mean ± SE |
| --- | --- | --- | --- | --- |
| Vehicle DMSO (1%) | 87.77 | 91.63 | 95.00 | 91.47±2.09 |
| DADS (1.0mM) | 47.54 | 44.94 | 35.73 | 42.74±3.59 |
| BM-FP (µg Total Phenol / mL) |  |  |  |  |
|  |  |  |  |  |
| 5 | 183.75 | 130.13 | 145.84 | 153.24±15.93 |
| 10 | 146.64 | 124.64 | 138.34 | 136.54±6.42 |
| 20 | 94.53 | 92.67 | 105.58 | 97.59±4.03 |
| 40 | 42.94 | 54.05 | 57.71 | 51.57±4.45 |
| 80 | 34.55 | 47.63 | 48.17 | 43.45±4.46 |

***ST-3H. Effect of BTM-B* *against MDA-MB-468 cells viability***

|  | Batch-1 | Batch-2 | Batch-3 | Mean ± SE |
| --- | --- | --- | --- | --- |
| Vehicle Ethanol (1%) | 102.95 | 97.94 | 95.38 | 98.76±2.23 |
| DADS (1mM) | 47.54 | 44.94 | 35.73 | 42.74±3.59 |
| BM-BP (µg Total Phenol / mL) |  |  |  |  |
| 5 | 100.08 | 132.23 | 130.83 | 121.05±10.50 |
| 10 | 105.34 | 138.30 | 139.86 | 127.83±11.27 |
| 20 | 107.04 | 133.84 | 138.13 | 126.34±9.74 |
| 40 | 104.14 | 127.18 | 133.77 | 121.70±8.99 |
| 80 | 47.55 | 50.81 | 56.80 | 51.72±2.71 |

***ST-3I. Effect of BTM-F* *against MDA-MB-231 cells viability***

|  | Batch-1 | Batch-2 | Batch-3 | Mean ± SE |
| --- | --- | --- | --- | --- |
| Vehicle DMSO (1%) | 95.01 | 91.38 | 93.85 | 93.41±1.07 |
| DADS (1.0mM) | 60.38 | 74.59 | 72.31 | 69.09±4.41 |
| BM-FP (µg Total Phenol / mL) |  |  |  |  |
| 5 | 119.45 | 117.13 | 113.04 | 116.54±1.88 |
| 10 | 113.11 | 105.91 | 93.18 | 104.07±5.83 |
| 20 | 96.55 | 90.31 | 73.12 | 86.66±7.01 |
| 40 | 82.93 | 71.64 | 66.62 | 73.73±4.83 |
| 80 | 47.23 | 48.56 | 32.22 | 42.67±5.24 |

***ST-3J. Effect of BTM-B* *against MDA-MB-231 cells viability***

|  | Batch-1 | Batch-2 | Batch-3 | Mean ± SE |
| --- | --- | --- | --- | --- |
| Vehicle Ethanol (1%) | 95.79 | 99.33 | 95.29 | 96.81±1.27 |
| DADS (1mM) | 60.38 | 74.59 | 72.31 | 69.09±4.41 |
| BM-BP (µg Total Phenol / mL) |  |  |  |  |
| 5 | 100.00 | 107.39 | 112.95 | 106.78±3.76 |
| 10 | 97.86 | 105.61 | 100.58 | 101.35±2.27 |
| 20 | 85.06 | 90.81 | 95.24 | 90.37±2.95 |
| 40 | 70.47 | 76.32 | 80.45 | 75.75±2.90 |
| 80 | 33.60 | 35.28 | 45.96 | 38.28±3.88 |

***ST-3K. Effect of BTM-F* *against MCF-7cells viability***

|  | Batch-1 | Batch-2 | Batch-3 | Mean ± SE |
| --- | --- | --- | --- | --- |
| Vehicle DMSO (1%) | 88.32 | 77.98 | 93.17 | 86.49±4.49 |
| DADS (1.0mM) | 47.54 | 44.94 | 35.73 | 42.74±3.59 |
| BM-FP (µg Total Phenol / mL) |  |  |  |  |
| 5 | 75.38 | 83.18 | 83.28 | 80.61±2.62 |
| 10 | 58.51 | 59.23 | 63.40 | 60.38±1.53 |
| 20 | 51.35 | 50.89 | 57.30 | 53.18±2.07 |
| 40 | 53.57 | 33.35 | 43.73 | 43.55±5.84 |
| 80 | 52.25 | 36.89 | 45.26 | 44.80±4.45 |

***ST-3L. Effect of BTM-B* *against MCF-7 cells viability***

|  | Batch-1 | Batch-2 | Batch-3 | Mean ± SE |
| --- | --- | --- | --- | --- |
| Vehicle Ethanol (1%) | 93.69 | 100.91 | 100.73 | 98.44±2.38 |
| DADS (1mM) | 47.54 | 44.94 | 35.73 | 42.74±3.59 |
| BM-BP (µg Total Phenol / mL) |  |  |  |  |
| 5 | 92.92 | 98.35 | 86.63 | 92.63±3.39 |
| 10 | 88.78 | 86.36 | 73.48 | 82.87±4.76 |
| 20 | 88.02 | 71.54 | 57.97 | 72.51±8.70 |
| 40 | 69.56 | 61.54 | 51.56 | 60.89±5.21 |
| 80 | 42.97 | 49.64 | 35.33 | 42.64±4.14 |

***ST-3M. Effect of BTM-F* *against BT-474cells viability***

|  | Batch-1 | Batch-2 | Batch-3 | Mean ± SE |
| --- | --- | --- | --- | --- |
| Vehicle DMSO (1%) | 96.91 | 102.39 | 97.88 | 99.06±1.69 |
| DADS (1.0mM) | 76.91 | 75.57 | 72.31 | 74.93±1.37 |
| BM-FP (µg Total Phenol / mL) |  |  |  |  |
| 5 | 109.49 | 117.03 | 115.35 | 113.95±2.29 |
| 10 | 90.40 | 85.89 | 96.09 | 90.79±2.95 |
| 20 | 70.59 | 84.73 | 71.23 | 75.52±4.61 |
| 40 | 52.22 | 40.61 | 37.97 | 43.60±4.38 |
| 80 | 29.47 | 22.46 | 21.49 | 24.47±2.52 |

***ST-3N. Effect of BTM-B* *against BT-474 cells viability***

|  | Batch-1 | Batch-2 | Batch-3 | Mean ± SE |
| --- | --- | --- | --- | --- |
| Vehicle Ethanol (1%) | 94.32 | 99.13 | 92.38 | 95.28±2.01 |
| DADS (1mM) | 76.91 | 75.57 | 72.31 | 74.93±1.37 |
| BM-BP (µg Total Phenol / mL) |  |  |  |  |
| 5 | 101.23 | 116.74 | 118.13 | 112.03±5.42 |
| 10 | 105.91 | 110.38 | 103.75 | 106.68±1.95 |
| 20 | 68.15 | 83.25 | 90.90 | 80.77±6.69 |
| 40 | 56.84 | 59.68 | 62.18 | 59.57±1.54 |
|  |  |  |  |  |
| 80 | 47.76 | 42.26 | 49.95 | 46.65±2.29 |

***ST-3O. Effect of BTM-F* *against BEAS-2B cells viability***

|  | Batch-1 | Batch-2 | Batch-3 | Mean ± SE |
| --- | --- | --- | --- | --- |
| Vehicle DMSO (1%) | 63.11 | 60.07 | 69.33 | 64.17±2.73 |
| DADS (1mM) | 68.89 | 61.05 | 49.80 | 59.91±5.54 |
| BM-FP (µg Total Phenol / mL) |  |  |  |  |
| 5 | 89.00 | 59.48 | 94.92 | 81.13±10.98 |
| 10 | 62.07 | 47.49 | 81.11 | 63.56±9.74 |
| 20 | 51.38 | 29.19 | 70.61 | 50.39±11.98 |
| 40 | 41.54 | 39.09 | 55.70 | 34.04±5.18 |
| 80 | 39.37 | 30.29 | 48.56 | 39.41±5.28 |

***ST-3P. Effect of BTM-B*** ***against BEAS-2B cells viability***

|  | Batch-1 | Batch-2 | Batch-3 | Mean ± SE |
| --- | --- | --- | --- | --- |
| Vehicle Ethanol (1%) | 97.99 | 97.77 | 96.13 | 97.30±0.59 |
| DADS (1mM) | 68.89 | 61.05 | 49.80 | 59.91±5.54 |
| BM-BP (µg Total Phenol / mL) |  |  |  |  |
| 5 | 91.37 | 72.49 | 62.50 | 75.45±8.47 |
| 10 | 89.20 | 56.15 | 52.38 | 65.91±11.71 |
| 20 | 83.25 | 46.19 | 45.86 | 58.43±12.42 |
| 40 | 22.00 | 44.89 | 23.79 | 30.23±7.36 |
| 80 | 8.83 | 12.09 | 10.98 | 10.63±0.96 |

***ST-3Q. Effect of BTM-F* *against HaCaT cells viability***

|  | Batch-1 | Batch-2 | Batch-3 | Mean ± SE |
| --- | --- | --- | --- | --- |
| Vehicle Ethanol (1%) | 77.42 | 76.75 | 81.47 | 78.55±1.48 |
| DADS (1mM) | 33.52 | 37.03 | 31.65 | 34.07±1.58 |
| BM-FP (µg Total Phenol / mL) |  |  |  |  |
| 5 | 108.22 | 108.74 | 107.52 | 108.16±0.36 |
| 10 | 93.94 | 90.30 | 107.11 | 97.12±5.11 |
| 20 | 48.12 | 51.98 | 97.65 | 65.92±15.92 |
| 40 | 32.91 | 33.98 | 47.60 | 38.16±4.73 |
| 80 | 51.01 | 49.38 | 31.13 | 43.84±6.38 |

***ST-3P. Effect of BTM-B against HaCaT cells viability***

|  | Batch-1 | Batch-2 | Batch-3 | Mean ± SE |
| --- | --- | --- | --- | --- |
| Vehicle Ethanol (1%) | 98.02 | 95.95 | 97.18 | 97.05±0.60 |
| DADS (1mM) | 33.52 | 37.03 | 31.65 | 34.07±1.58 |
| BM-BP (µg Total Phenol / mL) |  |  |  |  |
| 5 | 109.00 | 104.55 | 107.96 | 107.17±1.35 |
| 10 | 108.22 | 106.81 | 96.20 | 103.75±3.80 |
| 20 | 104.92 | 102.18 | 101.51 | 102.87±1.05 |
| 40 | 99.21 | 85.11 | 60.07 | 81.46±11.46 |
| 80 | 46.78 | 24.30 | 33.47 | 34.85±6.53 |

ST-4A. UPLC-QTOF-MS analysis of BTM-F

| **Peak** | **Retention Time (min)** | **Peak Area** | **Molecular Mass (amu) (Experimental)** | **Molecular Mass (amu)**  **(Theoretical)** | **Error** | **Common Name** | **IUPAC Name** | **Molecular formula** |
| --- | --- | --- | --- | --- | --- | --- | --- | --- |
| 1 | 1.440 | 5305382  (1.31 %) | 174.0169 | 174.0164 | 2.76 | cis-Aconitic acid | (Z)-prop-1-ene-1,2,3-tricarboxylic acid | C_6_ H_6_ O_6_ |
| 2 | 1.878 | 1947584 | 138.0321 | 138.0317 | 3.12 | 4-Hydroxybenzoic acid | 4-Hydroxybenzoic acid | C_7_ H_6_ O_3_ |
| 3 | 1.944 | 7832000 | 219.1113 | 219.1107 | 3.05 | D-Pantothenic acid | 3-[[(2R)-2,4-dihydroxy-3,3-dimethylbutanoyl]amino]propanoic acid | C_9_ H_17_ N O_5_ |
| 4 | 4.662 | 3573845 | 180.0428 | 180.0423 | 2.91 | Caffeic acid | (E)-3-(3,4-dihydroxyphenyl)prop-2-enoic acid | C_9_ H_8_ O_4_ |
| 5 | 5.558 | 5345043 | 165.0793 | 165.0790 | 2.10 | L-Phenylalanine | (2S)-2-amino-3-phenylpropanoic acid | C_9_ H_11_ N O_2_ |
| 6 | 10.357 | 2218724 | 244.1314 | 244.1311 | 1.36 | New Compound |  | C_12_ H_20_ O_5_ |
| 7 | 10.186 | 1427514 | 386.1218 | 386.1213 | 1.18 | Sinapoyl glycoside | [(3R,4S,5S,6R)-3,4,5-trihydroxy-6-(hydroxymethyl)oxan-2-yl] (E)-3-(4-hydroxy-3,5-dimethoxyphenyl)prop-2-enoate | C_17_ H_22_ O_10_ |
| 8 | 11.101 | 1957977 | 462.0806 | 462.0798 | 1.59 | Kaempferol 3-glucuronide | (2S,3S,4S,5R,6S)-6-[5,7-dihydroxy-2-(4-hydroxyphenyl)-4-oxochromen-3-yl]oxy-3,4,5-trihydroxyoxane-2-carboxylic acid | C_21_ H_18_ O_12_ |
| 9 | 11.513 | 5420611 | 166.0634 | 166.0630 | 2.29 | Dihydro-3-coumaric acid | 3-(3-hydroxyphenyl)propanoic acid | C_9_ H_10_ O_3_ |
| 10 | 16.082 | 16319031 | 178.0634 | 178.0630 | 2.46 | (R)-(-)-Mellein | 8-hydroxy-3-methyl-3,4-dihydroisochromen-1-one | C_10_ H_10_ O_3_ |
| 11 | 16.217 | 3011856 | 194.0582 | 194.0579 | 1.49 | Ferulic acid | (E)-3-(4-hydroxy-3-methoxyphenyl)prop-2-enoic acid | C_10_ H_10_ O_4_ |
| 12 | 16.695 | 1099872 | 469.2209 | 469.2213 - | 0.81 | N′, N″-dicaffeoylspermidine | 1099872 | C_25_ H_31_ N_3_ O_6_ |
| 13 | 17.517 | 21147950 | 448.1021 | 448.1006 | 3.52 | Naringenin-7-O-β-DGlucuronide | (2S,3S,4S,5R,6S)-3,4,5-trihydroxy-6-[[5-hydroxy-2-(4-hydroxyphenyl)-4-oxo-2,3-dihydrochromen-7-yl]oxy]oxane-2-carboxylic acid | C_21_ H_20_ O_11_ |
| 14 | 20.313 | 2388000 | 396.0854 | 396.0845 | 2.29 | Apigenin Triacetate | [4-(5,7-diacetyloxy-4-oxochromen-2-yl)phenyl] acetate | C_21_ H_16_ O_8_ |
| 15 | 20.335 | 3578698 | 594.1597 | 594.1585 | 2.04 | Apigenin 7,4'-diglucoside | 5-hydroxy-7-[(2S,5S,6S)-3,4,5-trihydroxy-6-(hydroxymethyl)oxan-2-yl]oxy-2-[4-[(2S,5S)-3,4,5-trihydroxy-6-(hydroxymethyl)oxan-2-yl]oxyphenyl]chromen-4-one | C_27_ H_30_ O_15_ |
| 16 | 20.335 | 3120158 | 640.1654 | 640.1639 | 2.32 | Rhamnetin 3-sophoroside | 3-[4,5-dihydroxy-6-(hydroxymethyl)-3-[3,4,5-trihydroxy-6-(hydroxymethyl)oxan-2-yl]oxyoxan-2-yl]oxy-2-(3,4-dihydroxyphenyl)-5-hydroxy-7-methoxychromen-4-one | C_28_ H_32_ O_17_ |
| 17 | 20.467 | 4265715 | 208.0739 | 208.0736 | 1.83 | Dimethylcaffeic acid | (E)-3-(3,4-dimethoxyphenyl)prop-2-enoic acid | C_11_ H_12_ O_4_ |
| 18 | 20.489 | 16665390 | 432.1050 | 432.1056 | -1.41 | Apigenin 7-glucoside | 5-hydroxy-2-(4-hydroxyphenyl)-7-[(2S,3R,4S,5S,6R)-3,4,5-trihydroxy-6-(hydroxymethyl)oxan-2-yl]oxychromen-4-one | C_21_ H_20_ O_10_ |
| 19 | 20.489 | 1191662 | 478.1121 | 478.1111 | 2.02 | Isorhamnetin 3-galactoside | 5,7-dihydroxy-2-(4-hydroxy-3-methoxyphenyl)-3-[3,4,5-trihydroxy-6-(hydroxymethyl)oxan-2-yl]oxychromen-4-one | C_22_ H_22_ O_12_ |
| 20 | 21.941 | 287027768 | 444.1075 | 444.1056 | 4.24 | Formononetin 7-Oglucuronide | (2S,3S,4S,5R,6S)-3,4,5-trihydroxy-6-[3-(4-methoxyphenyl)-4-oxochromen-7-yl]oxyoxane-2-carboxylic acid | C_22_ H_20_ O_10_ |
| 21 | 22.138 | 1138573 | 386.0998 | 386.1002 | -1.03 | Diferulic acid | (E)-3-[3-[5-[(E)-2-carboxyethenyl]-2-hydroxy-3-methoxyphenyl]-4-hydroxy-5-methoxyphenyl]prop-2-enoic acid | C_20_ H_18_ O_8_ |
| 22 | 22.204 | 1140478 | 330.0743 | 330.0740 | 1.16 | Tricin | 5,7-dihydroxy-2-(4-hydroxy-3,5-dimethoxyphenyl)chromen-4-one | C_17_ H_14_ O_7_ |
| 23 | 25.699 | 6407180 | 442.0920 | 442.0900 | 4.64 | Catechin 7-O-gallate | [(2R,3S)-2-(3,4-dihydroxyphenyl)-3,5-dihydroxy-3,4-dihydro-2H-chromen-7-yl] 3,4,5-trihydroxybenzoate | C_22_ H_18_ O_10_ |

ST-4B. UPLC-QTOF-MS analysis of BTM-B.

| **Peak** | **Retention Time (min)** | **Peak Area** | **Molecular Mass (amu) (Experimental)** | **Molecular Mass (amu)**  **(Theoretical)** | **Error** | **Common Name** | **IUPAC Name** | **Molecular formula** |
| --- | --- | --- | --- | --- | --- | --- | --- | --- |
| 1 | 1.479 | 1124941 | 174.0169 | 174.0164 | 2.90 | cis-Aconitic acid | (Z)-prop-1-ene-1,2,3-tricarboxylic acid | C_6_ H_6_ O_6_ |
| 2 | 2.429 | 1180351 | 168.0426 | 168.0423 | 2.32 | Vanillic acid | 4-hydroxy-3-methoxybenzoic acid | C_8_ H_8_ O_4_ |
| 3 | 3.755 | 1207932 | 154.0270 | 154.0266 | 2.55 | Protocatehuic acid | 3,4-Dihydroxybenzoic acid | C_7_ H_6_ O_4_ |
| 4 | 6.707 | 14535018 | 166.0630 | 166.0630 - | 0.19 | Dihydro-3-coumaric acid | 3-(3-hydroxyphenyl)propanoic acid | C_9_ H_10_ O_3_ |
| 5 | 10.287 | 1990301 | 208.0742 | 208.0736 | 3.01 | Dimethylcaffeic acid | (E)-3-(3,4-dimethoxyphenyl)prop-2-enoic acid | C_11_ H_12_ O_4_ |
| 6 | 10.683 | 9753767 | 386.1016 | 386.1002 | 3.83 | Diferulic acid | (E)-3-[3-[5-[(E)-2-carboxyethenyl]-2-hydroxy-3-methoxyphenyl]-4-hydroxy-5-methoxyphenyl]prop-2-enoic acid | C_20_ H_18_ O_8_ |
| 7 | 13.061 | 7132480 | 244.1319 | 244.1311 | 3.27 | New Compound |  | C12 H20 O5 |
| 8 | 13.061 | 3552760 | 270.0904 | 270.0892 | 4.46 | Dihydroformononetin | 7-hydroxy-3-(4-methoxyphenyl)-2,3-dihydrochromen-4-one | C_16_ H_14_ O_4_ |
| 9 | 17.103 | 3036093 | 224.0688 | 224.0685 | 1.65 | Sinapic acid | (E)-3-(4-hydroxy-3,5-dimethoxyphenyl)prop-2-enoic acid | C_11_ H_12_ O_5_ |
| 10 | 20.002 | 14510438 | 178.0635 | 178.0630 | 2.73 | (R)-(-)-Mellein | 8-hydroxy-3-methyl-3,4-dihydroisochromen-1-one | C_10_ H_10_ O_3_ |
| 11 | 20.458 | 2112608 | 432.1075 | 432.1056 | 4.28 | Apigenin 7-glucoside | 5-hydroxy-2-(4-hydroxyphenyl)-7-[(2S,3R,4S,5S,6R)-3,4,5-trihydroxy-6-(hydroxymethyl)oxan-2-yl]oxychromen-4-one | C_21_ H_20_ O_10_ |
| 12 | 22.617 | 1161399 | 270.0533 | 270.0528 | 1.83 | Apigenin | 5,7-dihydroxy-2-(4-hydroxyphenyl)chromen-4-one | C_15_ H_10_ O_5_ |
| 13 | 22.907 | 2732393 | 330.0750 | 330.0740 | 3.11 | Tricin | 5,7-dihydroxy-2-(4-hydroxy-3,5-dimethoxyphenyl)chromen-4-one | C_17_ H_14_ O_7_ |

**Supplemental Figure 1A. Elution profile of BTM-F by Using UPLC-QTOF.**


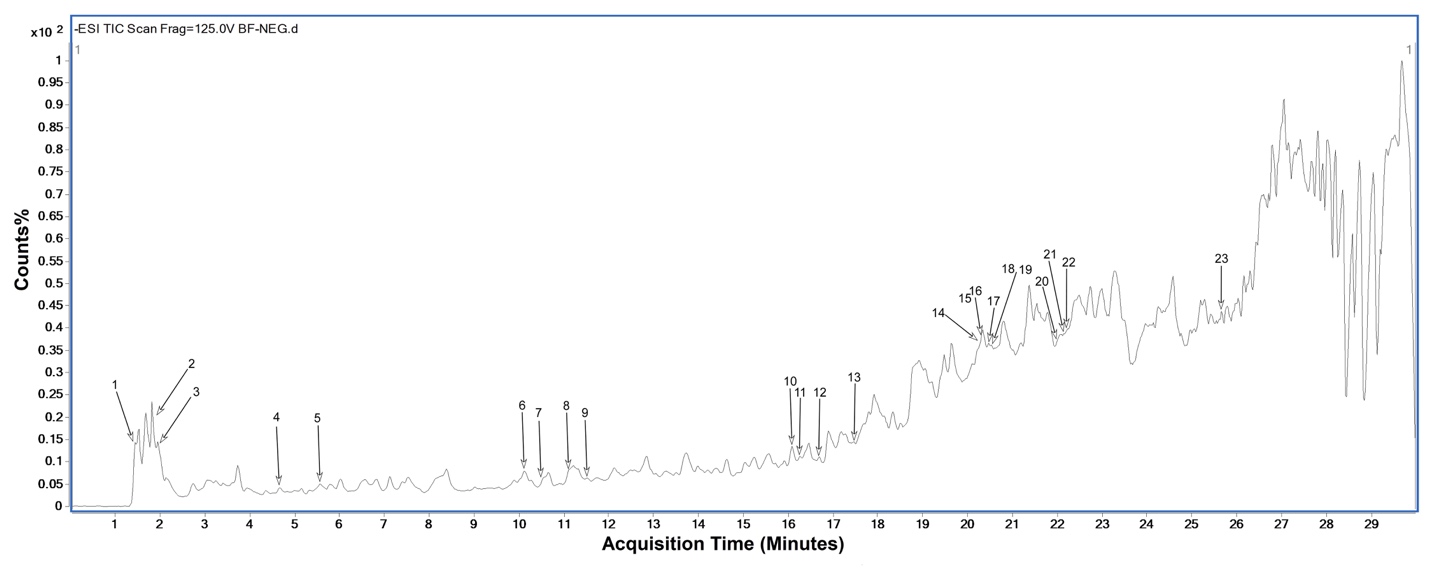


**Supplemental figure 1A. Analysis of BTM-F using UPLC-QTOF-MS**: In order to identify various metabolites, present in the BTM-F, UPLC-QTOF-MS was carried out as detailed in methods. Elution profile of BTM-F.

**Supplemental Figure 1B. Elution profile of BTM-B by Using UPLC-QTOF.**
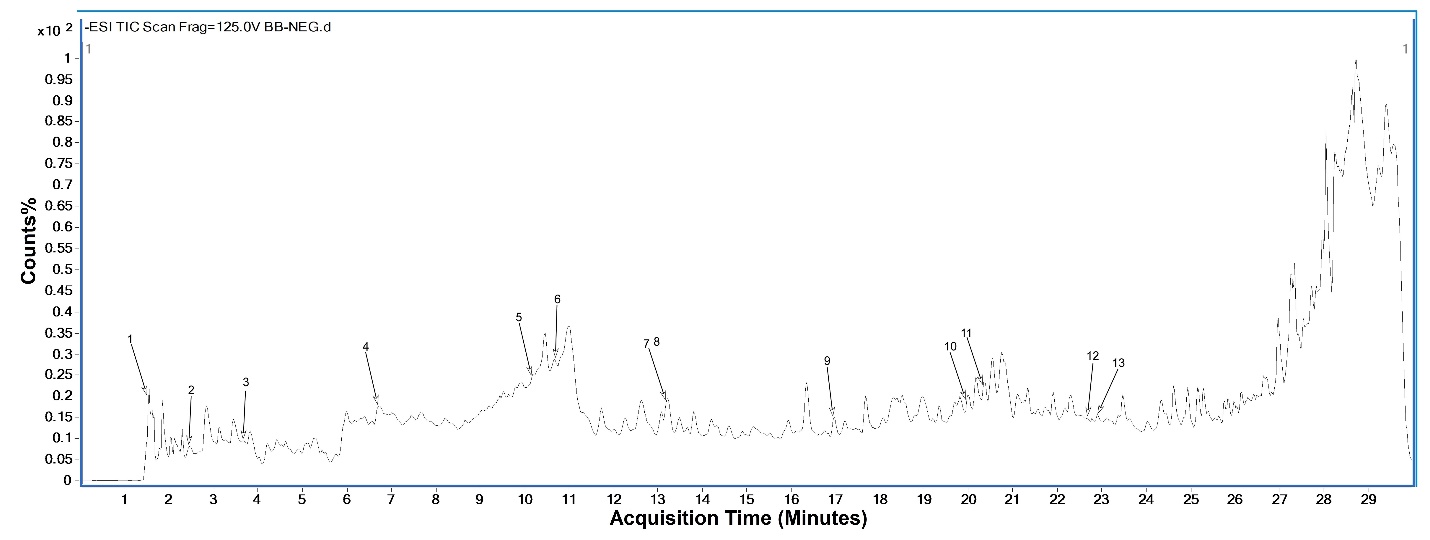


**Supplemental figure 1B. Analysis of BTM-BP using UPLC-QTOF-MS**: In order to identify various metabolites, present in the BTM-B, UPLC-QTOF-MS was carried out as detailed in methods. Elution profile of BTM-B.
